# Pathogen-selective killing by guanylate-binding proteins as a molecular mechanism leading to inflammasome signaling

**DOI:** 10.1038/s41467-022-32127-0

**Published:** 2022-07-29

**Authors:** Shouya Feng, Daniel Enosi Tuipulotu, Abhimanu Pandey, Weidong Jing, Cheng Shen, Chinh Ngo, Melkamu B. Tessema, Fei-Ju Li, Daniel Fox, Anukriti Mathur, Anyang Zhao, Runli Wang, Klaus Pfeffer, Daniel Degrandi, Masahiro Yamamoto, Patrick C. Reading, Gaetan Burgio, Si Ming Man

**Affiliations:** 1 Division of Immunology and Infectious Disease, The John Curtin School of Medical Research, The Australian National University, Canberra, ACT Australia; 2 Department of Microbiology and Immunology, The University of Melbourne, The Peter Doherty Institute for Infection and Immunity, Melbourne, VIC Australia; 3 Institute of Medical Microbiology and Hospital Hygiene, Heinrich-Heine-University Düsseldorf, Düsseldorf, Germany; 4 Department of Immunoparasitology, Research Institute for Microbial Diseases, Osaka University, Osaka, Japan; 5 Laboratory of Immunoparasitology, WPI Immunology Frontier Research Center, Osaka University, Osaka, Japan; 6 WHO Collaborating Centre for Reference and Research on Influenza, Victorian Infectious Diseases Reference Laboratory, The Peter Doherty Institute for Infection and Immunity, Melbourne, VIC Australia

**Keywords:** Inflammation, Pattern recognition receptors, Monocytes and macrophages, Infection

## Abstract

Inflammasomes are cytosolic signaling complexes capable of sensing microbial ligands to trigger inflammation and cell death responses. Here, we show that guanylate-binding proteins (GBPs) mediate pathogen-selective inflammasome activation. We show that mouse GBP1 and GBP3 are specifically required for inflammasome activation during infection with the cytosolic bacterium *Francisella novicida*. We show that the selectivity of mouse GBP1 and GBP3 derives from a region within the N-terminal domain containing charged and hydrophobic amino acids, which binds to and facilitates direct killing of *F. novicida* and *Neisseria meningitidis*, but not other bacteria or mammalian cells. This pathogen-selective recognition by this region of mouse GBP1 and GBP3 leads to pathogen membrane rupture and release of intracellular content for inflammasome sensing. Our results imply that GBPs discriminate between pathogens, confer activation of innate immunity, and provide a host-inspired roadmap for the design of synthetic antimicrobial peptides that may be of use against emerging and re-emerging pathogens.

## Abstract (web summary)

Guanylate-binding proteins (GBP) have a function in inflammasome formation and pathogen defence. Here the authors show that these GBP proteins are able to kill certain bacteria and promote selective inflammasome activation and that this is mediated by specific GBP protein regions.

## Introduction

Inflammasomes are cytosolic signaling complexes essential for the host defense against invading pathogens and contribute to the development of cancer, autoinflammatory and metabolic diseases^1,2^. Assembly of the inflammasome complex is initiated by inflammasome sensors, which include members of the nucleotide-binding domain, leucine-rich repeat containing protein (NLR) family, the AIM2-like receptor (ALR) family, and the Tripartite Motif (TRIM) family^3,4^. These sensors detect pathogen-associated molecular patterns (PAMPs), danger-associated molecular patterns (DAMPs) and homeostasis-altering molecular processes (HAMPs)^5,6^. Activated inflammasome sensors further recruit the cysteine protease caspase-1, either in the presence or absence of the adaptor protein apoptosis-associated speck-like protein containing a CARD (also known as ASC) to form a functional inflammasome complex^7^. Activated caspase-1 cleaves the pore-forming protein gasdermin D (GSDMD), and the pro-inflammatory cytokines pro-interleukin (IL)−1β and pro-IL-18 into their mature forms^8–10^. IL-1β and IL-18 escape through the pores formed by gasdermin D to trigger inflammation^11,12^. Further, the membrane disruptor protein called nerve injury-induced protein 1 (also known as NINJ1) oligomerizes to induce plasma membrane rupture and pyroptosis^13^, releasing potentially hundreds of inflammatory, signaling, and structural molecules from the cell.

Inflammasome sensors have pathogen-specificity which is determined by the ability of the inflammasome sensor to bind a specific ligand, to detect a more generic signal, or be directed by regulatory factors which present these signals to the inflammasome sensor^14–16^. For example, the NAIP inflammasome sensors can bind directly to flagellin or proteins of the Type III secretion system of bacteria to allow detection of pathogenic bacteria such as *Salmonella enterica* serovar Typhimurium (also known as *S*. Typhimurium)^17,18^. The inflammasome sensor NLRP3 responds to PAMPs and DAMPs indirectly by interpreting signals, including ion fluxes, organelle damage, cellular stress, or disassembly of the *trans*-Golgi network^19–25^.

The inflammasome sensor AIM2 binds specifically to dsDNA^26–29^, suggesting that it may sense any DNA-carrying microbes or host-derived DNA. However, AIM2 exhibits remarkable pathogen-selectivity and responds only to a small subset of pathogens, such as the bacterium *Francisella tularensis*, the virus mouse cytomegalovirus, the fungus *Aspergillus fumigatus*, and the parasites *Toxoplasma gondii* and *Plasmodium falciparum*^30–34^. This selectivity is, in part, determined by an array of regulatory factors which facilitates cytosolic presentation of DNA to AIM2. We and others have shown that IFN-inducible GTPases, including the guanylate-binding proteins (GBPs) and immunity-related GTPases (IRGs), can contribute to inflammasome responses^35–39^. Indeed, GBP2 primarily mediates activation of the inflammasome in response to the bacterial pathogens *Escherichia coli* and *Citrobacter rodentium*, whereas GBP2, GBP5, and IRGB10 license inflammasome activation in response to the bacterium *F. novicida* in mouse macrophages^35–39^. Further, GBP1 controls the replication of *S*. Typhimurium and *T. gondii* in human macrophages^40,41^, and can bind to lipopolysaccharide (LPS) of *S*. Typhimurium and *Shigella flexneri*, triggering activation of the caspase-4 inflammasome^42–45^. How each member of the GBP family, which comprises 11 GBPs in mice and 7 in humans, discriminates microbes and cooperates with one another to drive pathogen-selective inflammasome responses has remained largely unclear. The fact that human GBP1 can bind LPS suggests that this family of proteins might represent a class of mammalian cytosolic innate immune receptors.

Here, we took advantage of CRISPR-Cas9 gene editing technology to generate mice lacking GBPs encoded on the chromosome 3 genomic cluster (GBP1, GBP2, GBP3, GBP5, and GBP7) to comprehensively study the individual contributions of GBPs in inflammasome activation by *F. novicida*. In addition to GBP2 and GBP5, we show that GBP1 and GBP3 contribute to pathogen-selectivity towards *F. novicida*, mediating activation of the inflammasome. We show that recombinant full-length GBP1 is antimicrobial, of which the N-terminal globular domain of GBP1 and the corresponding region of GBP3 mediate the selective binding and killing of *F. novicida* and *N. meningitidis*. Therefore, we show that GBPs can dictate pathogen-selectivity leading to effective innate immune recognition and killing of pathogens.

## Results

### GBPs induce pathogen-selective inflammasome activation

Activation of the inflammasome pathway by Gram-negative bacteria, such as *F. novicida*, requires type I interferons (IFNs) (Supplementary Fig. 1a, b)^36,37,46–48^. Type I IFNs promote the expression of hundreds of IFN-inducible proteins, including GBPs, of which GBP1, 2, 3, 5, and 7 are clustered on chromosome 3 within the mouse genome. Indeed, primary mouse bone marrow-derived macrophages lacking the type I IFN receptors, or all 5 GBPs within the chromosome 3 cluster (called *Gbp*^chr3^-KO)^49^, had an impaired ability to undergo caspase-1 and GSDMD cleavage, secretion of IL-1β and IL-18, and cell death following *F. novicida* infection compared with wildtype (WT) BMDMs (Supplementary Fig. 1a, b)^36,37^.

**Fig. 1:**
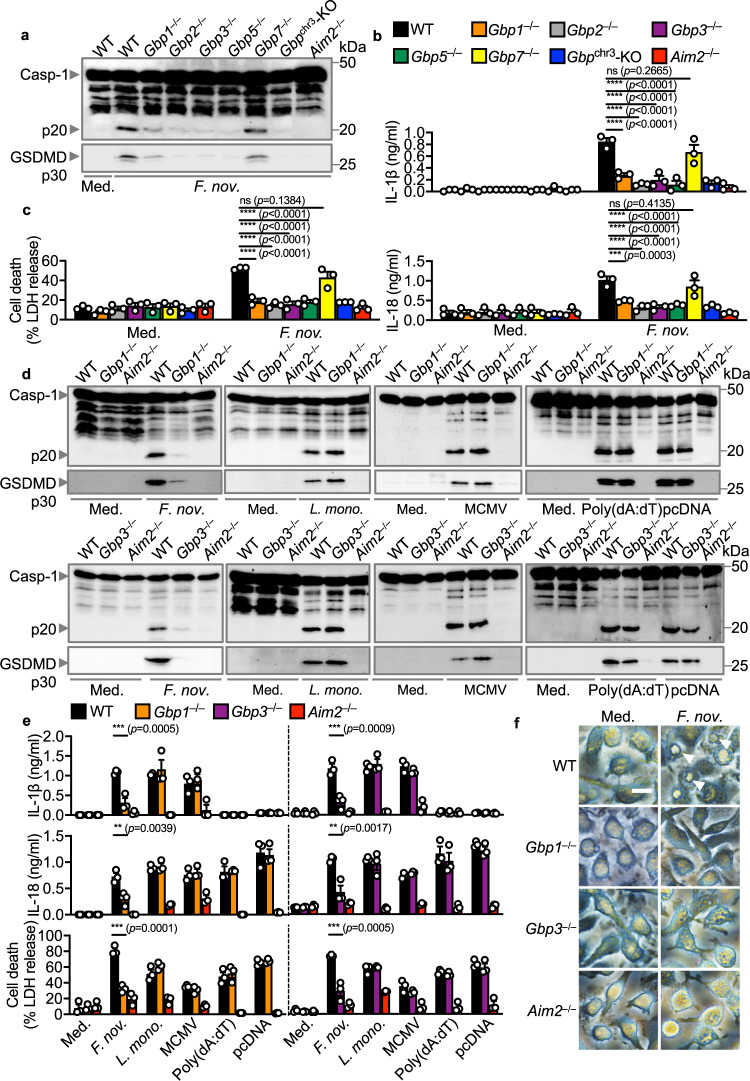
GBP1, GBP2, GBP3, and GBP5 are required for *F. novicida*-induced inflammasome activation. **a** Immunoblot analysis of caspase-1 (Casp-1) and gasdermin D (GSDMD) in WT, *Gbp1*^–/–^, *Gbp2*^–/–^, *Gbp3*^–/–^, *Gbp5*^–/–^, *Gbp7*^–/–^, *Gbp*^chr3^-KO, or *Aim2*^–/–^ BMDMs left untreated (Med.) or assessed after infection with *F. novicida* (MOI 100) for 10 h. **b** The release of IL-1β and IL-18 from BMDMs after treatment as in **a**. **c** The release of LDH from BMDMs after treatment as in **a**. **d** Immunoblot analysis of Casp-1 and GSDMD in WT, *Gbp1*^–/–^, *Gbp3*^–/–^ and *Aim2*^–/–^ BMDMs left untreated (Med.) or assessed after infection with *F. novicida* (MOI 100) for 10 h, infection with *L. monocytogenes* (MOI 100) for 20 h, infection with MCMV (MOI 10) for 10 h or transfection of poly(dA:dT) (5 µg/ml) and pcDNA (5 µg/mL) for 4 h. **e** The release of IL-1β, IL-18 and LDH from BMDMs after treatment as in **d**. **f** Light microscopy analysis of WT, *Gbp1*^–/–^, *Gbp3*^–/–^ or *Aim2*^–/–^ BMDMs left untreated (Med.) or following infection with *F. novicida* as in **d**. White arrows indicate pyroptotic cells. Each symbol represents an independent experiment (**b**, **c**, and **e**). Scale bar, 20 µm (**f**). ns no statistical significance; **P < 0.01; ***P < 0.001; ****P < 0.0001 (one-way ANOVA with Dunnett’s multiple-comparisons test (**b**, **c**, and **e**)). Data are representative of three independent experiments (**a**–**f**; mean and s.e.m. in **b**, **c**, and **e**). Source data are provided as a Source data file.

To comprehensively investigate the individual and relative contributions of GBPs in inflammasome activation, we used CRISPR-Cas9 technology to generate mouse strains lacking each of the five GBPs on the chromosome 3 locus, followed by validation at the genomic and protein level (Supplementary Fig. 2). We infected WT, *Gbp1*^–/–^, *Gbp2*^–/–^, *Gbp3*^–/–^, *Gbp5*^–/–^, *Gbp7*^–/–^ BMDMs with *F. novicida* and monitored for hallmarks of inflammasome activation. We observed that *Gbp1*^–/–^, *Gbp2*^–/–^*, Gbp3*^–/–^, and *Gbp5*^–/–^ BMDMs infected with *F. novicida* had an impaired ability to induce caspase-1 and GSDMD cleavage, secretion of IL-1β and IL-18, and cell death compared to WT BMDMs (Fig. 1a–c). However, *Gbp7*^–/–^ BMDMs responded normally to *F. novicida* infection (Fig. 1a–c). In addition to *Gbp2*^–/–^ and *Gbp5*^–/–^ BMDMs^36,37^, we found that *Gbp1*^–/–^ and *Gbp3*^–/–^ BMDMs had an impaired ability to generate ASC specks, a hallmark of inflammasome activation, in response to infection with *F. novicida* (Supplementary Fig. 1c), suggesting that four different GBPs, including GBP1 and GBP3, function to contribute to inflammasome activation.

*F. novicida* is a cytosolic bacterial pathogen that induces activation of the DNA sensor AIM2, leading to the assembly of the AIM2 inflammasome, suggesting a specificity of GBP1 and GBP3 towards activation of AIM2. However, WT, *Gbp1*^–/–^ and *Gbp3*^–/–^ BMDMs infected with the AIM2 activators *Listeria monocytogenes* or MCMV, or transfected with the dsDNA species poly(dA:dT) or pcDNA undergo similar levels of inflammasome activation (Fig. 1d–f, Supplementary Fig. 1d). The defective activation of inflammasome responses to *F. novicida* infection is not owing to differences in the phosphorylation of ERK and IκB or in the expression of genes encoding IL-1β, IL-6, IL-18, CXCL1 (also known as KC), TNF and IFN-β between WT, *Gbp1*^–/–^ and *Gbp3*^–/–^ BMDMs (Supplementary Fig. 3a–c). We also show that deletion of *Gbp1* and *Gbp3* did not induce any point mutation or indels in the exons of *Gbp2* or *Gbp5* (Supplementary Tables 1–2), and that deletion of *Gbp2* (using a sgRNA targeting exon 5 with >70% sequence overlap with *Gbp7*) did not induce any point mutation or indels in the exons of *Gbp7* (Supplementary Table 3). Further, the absence of GBP1 or GBP3 in BMDMs did not affect the gene or protein expression of the remaining GBPs on chromosome 3 (Supplementary Fig. 3d–f). These results suggest that the lack of GBP1 or GBP3 does not affect other major inflammatory pathways or the expression of other GBPs, and importantly, indicate that GBP1 and GBP3 are additional IFN-inducible factors potentially linking pathogen-selectivity and inflammasome activation towards *F. novicida* infection.

*Gbp2*^–/–^ BMDMs are defective in the activation of the LPS-sensing caspase-11 inflammasome in response to the bacterial pathogens *E. coli* and *C. rodentium*^39^. Unlike *Gbp2*^–/–^ BMDMs, *Gbp1*^–/–^ and *Gbp3*^–/–^ BMDMs responded normally to the caspase-11 activators *C. rodentium*, *E. coli*, and cytosolic LPS (Supplementary Fig. 4a–d), the NAIP-NLRC4 inflammasome trigger *S*. Typhimurium (Supplementary Fig. 5a–d), the NLRP3 triggers ATP and nigericin (Supplementary Fig. 5e–h), and the Pyrin inflammasome trigger, the supernatant of *Clostridium difficile* (Supplementary Fig. 5i–l). These results further suggest a specificity of GBP1 and GBP3 towards *F. novicida*.

**Fig. 2:**
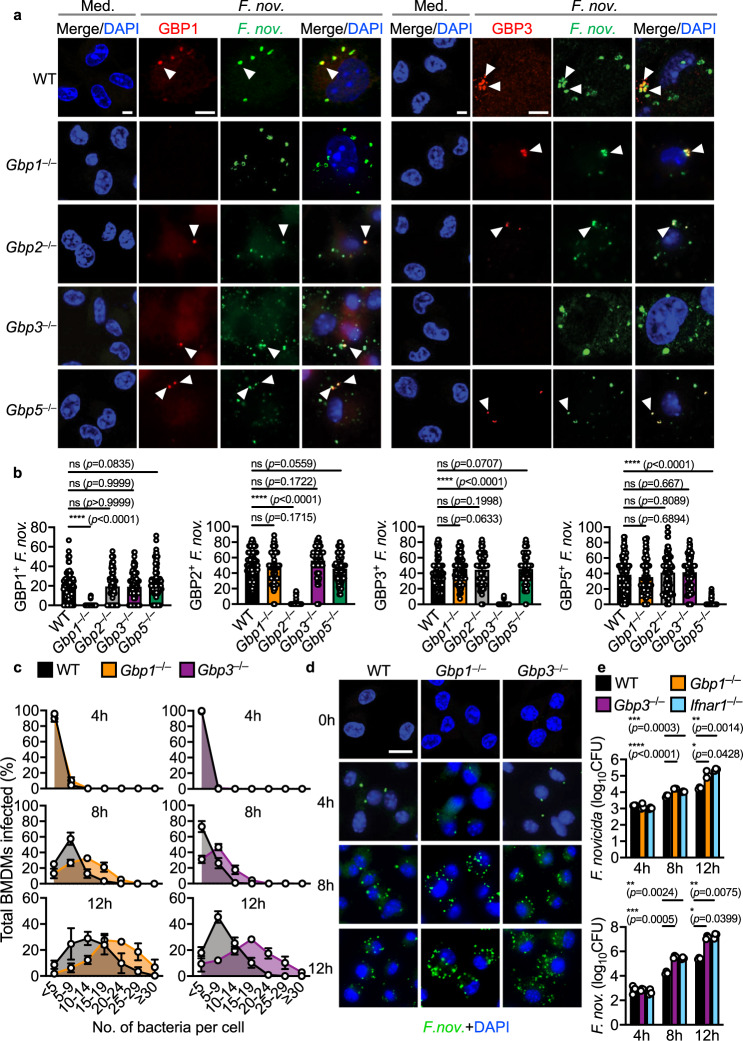
GBP1 and GBP3 target intracellular *F. novicida* and restrict its growth. **a** Confocal microscopy analysis of *F. novicida* (green) and GBP1 (red) or GBP3 (red) in WT, *Gbp1*^–/–^, *Gbp2*^–/–^, *Gbp3*^–/–^ and *Gbp5*^–/–^ BMDMs left untreated (Med.) or assessed 20 h after infection with *F. novicida* (MOI 20). White arrows indicate bacteria colocalized with GBP. **b** Quantitation of GBP1-, GBP2-, GBP3-, GBP5-positive *F. novicida* in WT, *Gbp1*^–/–^*, Gbp2*^–/–^, *Gbp3*^–/–^ and *Gbp5*^–/–^ BMDMs as treated in **a**. **c** The percentages of WT and *Gbp1*^–/–^ BMDMs (left) or WT and *Gbp3*^–/–^ BMDMs (right) harboring different number of bacteria. **d** Confocal microscopy analysis of *F. novicida* (green) and DNA (blue) in WT, *Gbp1*^–/–^ and *Gbp3*^–/–^ BMDMs 0, 4, 8, and 12 h after infection with *F. novicida* (MOI 25). **e** Recovery of *F. novicida* (as colony-forming units (CFU)) from WT, *Gbp1*^–/–^, *Gbp3*^–/–^ and *Ifnar1*^–/–^ BMDMs at 4, 8, or 12 h after infection with *F. novicida* (MOI 50). Scale bars, 7 µm (**a**) and 20 µm (**d**). ns no statistical significance; *P < 0.05, **P < 0.01; ***P < 0.001, ****P < 0.0001 (one-way ANOVA with Dunnett’s multiple-comparisons test (**b**, **e**)). Data are from one experiment representative of three independent experiments (**a**, **d**) or pooled from three independent experiments (**b**, **c**, and **e**, mean and s.e.m. in **b**, **c**, and **e**). Source data are provided as a Source data file.

The selectivity of certain GBPs towards *F. novicida* suggests that they may have pattern-recognition capabilities to target *F. novicida*. Immunofluorescence staining revealed that endogenous GBP1, GBP2, GBP3 and GBP5, the same GBPs required for inflammasome activation, co-localized with *F. novicida* in BMDMs, whereas GBP7 did not (Fig. 2a, Supplementary Fig. 6a, b). Recruitment of endogenous GBP1 and GBP3 to *F. novicida* was similar in *Gbp2*^–/–^ and *Gbp5*^–/–^ BMDMs compared to WT BMDMs (Fig. 2a, b). Conversely, the recruitment of endogenous GBP2 and GBP5 to *F. novicida* was similar in *Gbp1*^–/–^ and *Gbp3*^–/–^ BMDMs (Fig. 2b, Supplementary Fig. 6a). Although we observed that GBP1 and GBP3 specifically targets intracellular *F. novicida* to induce inflammasome activation in BMDMs, we did not address whether GBP1 and GBP3 are recruited to other cytosolic Gram-negative bacteria.

We further confirmed these findings in mouse lung epithelial LA-4 cells and found that IFN-γ-primed FLAG-tagged GBP1, GBP2, GBP3 or GBP5 co-localized with *F. novicida*, whereas GBP7 did not (Supplementary Fig. 7a). Moreover, we did not observe localization of FLAG-tagged GBP1, GBP2, GBP3, GBP5 or GBP7 to *F. novicida* in unprimed LA-4 cells (Supplementary Fig. 7a). Given that overexpressed GBP1 did not target to *F. novicida* within mouse lung epithelial cells (LA-4) in the absence of IFN-γ priming, this finding suggest that, under this condition, the intrinsic ability of GBP1 to target *F. novicida* is not sufficient and requires additional IFN-γ-inducible factors in order to target *F. novicida*.

To investigate whether GBPs restrict the replication of *F. novicida* in BMDMs, we infected WT, *Gbp1*^–/–^ and *Gbp3*^–/–^ BMDMs with *F. novicida* for 4, 8, or 12 h and observed increased number of intracellular *F. novicida* in *Gbp1*^–/–^ and *Gbp3*^–/–^ BMDMs over 12 h compared with WT BMDMs (Fig. 2c, d). Further, we quantified the number of intracellular bacteria by viable plate count and observed that *Gbp1*^–/–^, *Gbp3*^–/–^, and *Ifnar1*^–/–^ BMDMs harbored significantly more viable *F. novicida* over time compared to WT BMDMs (Fig. 2e). These data suggest that GBP1 and GBP3 were recruited to *F. novicida* and restricted replication of bacteria.

Our previous work revealed that GBPs encoded on the chromosome 3 cluster mediate the recruitment of IRGB10 to intracellular *F. novicida*^38^. To investigate whether individual GBPs are responsible for IRGB10 recruitment, we overexpressed IRGB10 and one GBP at a time (GBP1, GBP2, GBP3, GBP5 or GBP7) in LA-4 cells followed by infection with *F. novicida*. IRGB10 did not recruit to *F. novicida* in LA-4 cells (Supplementary Fig. 7b). These data suggest that a single GBP is insufficient to induce the recruitment of IRGB10 to intracellular *F. novicida*.

### GBP1 induces pathogen-selective bacteriolysis

Human GBP1 has been shown to bind to LPS of *S*. Typhimurium and *S. flexneri*, which drives the recruitment of several other GBPs to induce activation of caspase-4 in HeLa epithelial cells^42–45^. However, our data showed that mouse GBP1 does not contribute to inflammasome activation in response to either *S*. Typhimurium or *E. coli*, but instead targeted and promoted inflammasome activation to *F. novicida*. These data suggest that mGBP1 and hGBP1 differ in their biological activity. To determine whether mGBP1 can induce bacteriolysis in vitro we recombinantly expressed full-length mGBP1 and investigated whether it can kill *F. novicida* and *E. coli*. We observed that mGBP1 killed *F. novicida* in a dose-dependent manner, but not *E. coli* (Supplementary Fig. 8a). We also incubated *F. novicida* and *E. coli* with mGBP1 in the presence of the membrane-impermeable dye SYTOX and observed that mGBP1 localized to and induced uptake of SYTOX in *F. novicida*, whereas this did not occur with *E. coli* (Supplementary Fig. 8b). Furthermore, we used scanning electron microscopy (SEM) to investigate the membrane integrity and ultrastructure of *F. novicida* following treatment with mGBP1. We observed disruption of the membrane integrity, membrane rupture, and expulsion of intracellular content in bacteria treated with mGBP1 (Supplementary Fig. 8c).

**Fig. 3:**
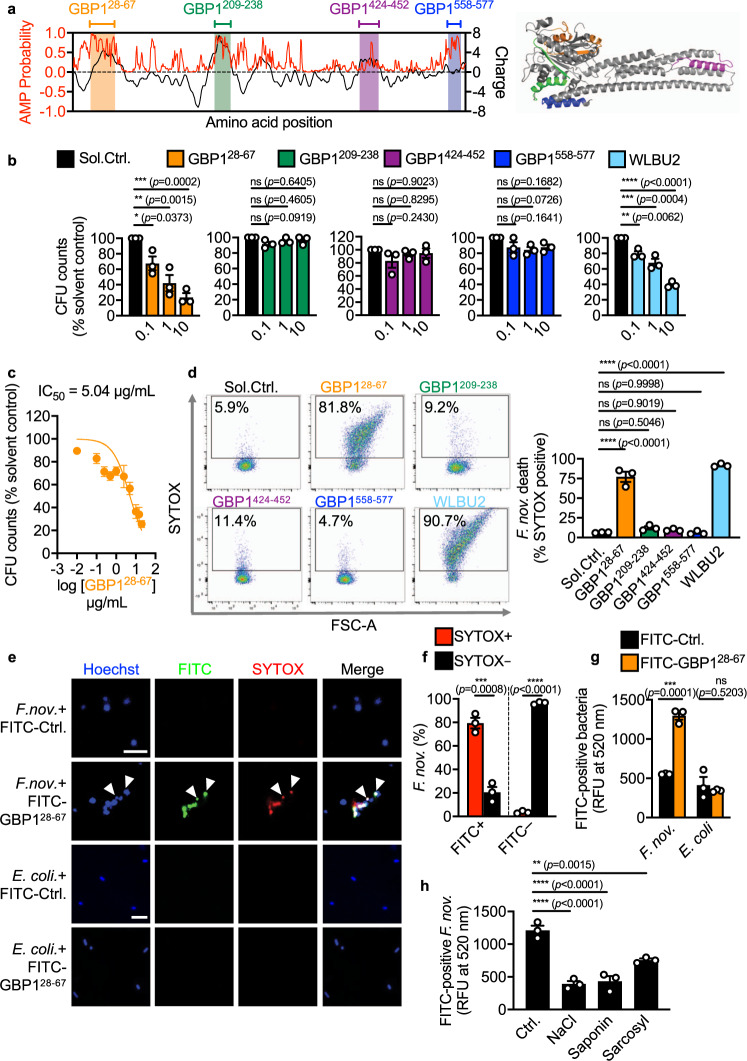
GBP1 peptide binds to and kills *F. novicida*. **a** Analysis of AMP probability (red) and charge (black) for the mouse GBP1 protein sequence and illustration of the location of putative antimicrobial stretches within the predicted mouse GBP1 structure. **b** Viability of *F. novicida* [*F. nov*., as percentage of CFU in relation to solvent control (Sol.Ctrl.)] assessed 6 h after incubation with GBP1^28–67^, GBP1^209–238^, GBP1^424–452^, GBP1^558–577^ or WLBU2 at 0.1, 1 or 10 μg/mL. **c** Viability of *F. nov*. following incubation with GBP1^28–67^ for 6 h over a concentration range (0.01–20 µg/mL) to determine the half maximal inhibitory concentration (IC_50_). **d** Flow cytometric analysis (left) and quantitation of flow cytometry plots (right) of SYTOX stained *F. novicida* treated with Sol.Ctrl. or 100 μg/mL of GBP1^28–67^, GBP1^209–238^, GBP1^424–452^, GBP1^558–577^ or WLBU2 for 12 h. **e** Confocal microscopy analysis of Hoechst-stained total bacteria (blue), FITC-GBP1^28–67^ (green) and SYTOX (red) in *F. novicida* or *E. coli* treated with 10 µg/mL FITC-GBP1^28–67^ or FITC-control peptide for 6 h. White arrows indicate dead bacteria covered with FITC-GBP1^28–67^. **f** Flow cytometric quantitation of SYTOX stained *F. novicida* treated with 10 µg/mL of FITC-GBP1^28–67^ for 6 h. **g** Quantitation of FITC-GBP1^28–67^ bound to *F. novicida* and *E. coli* after 1 h incubation with 10 µg/mL of either FITC- GBP1^28–67^ or a FITC-control peptide (in relative fluorescence units, RFU). **h** Quantitation of the effect of 1 M NaCl, 0.01% saponin or 0.08% sarcosyl on FITC-GBP1^28–67^ binding to *F. novicida*. Scale bar, 5 µm (**e**). ns no statistical significance; *P < 0.05, **P < 0.01; ***P < 0.001, ****P < 0.0001 (one-way ANOVA with Dunnett’s multiple-comparisons test (**b**, **d**, **h**), two-tailed *t*-test (**f**, **g**). Data are representative of three independent experiments (**b**–**h**; mean and s.e.m. in **b**–**d**, **f**–**h**). Source data are provided as a Source data file.

The recruitment of mouse GBP1 to *F. novicida* suggests that this protein may carry features which might bind and lyse *F. novicida*. Bioinformatic-assisted analysis revealed four stretches of amino acid sequences found across the globular head (amino acid position 1-309) and alpha-helical domains (amino acid position 310-589) of the GBP1 protein bearing a high antimicrobial property (AMP) probability score and charge (Fig. 3a). These peptides were designated GBP1^28–67^, GBP1^209–238^, GBP1^424–452^, GBP1^558–577^ (where the superscripted number indicates the amino acid position) (Supplementary Table 5). We synthesized these peptides and investigated their ability to kill *F. novicida*, compared to a known antimicrobial peptide, WLBU2^50^. Remarkably, only GBP1^28–67^ and WLBU2 exhibited, in a dose-dependent manner, antibacterial activity against *F. novicida* (Fig. 3b). The half maximal inhibitory concentration (also known as IC_50_) of GBP1^28–67^ was 5.04 μg/mL (Fig. 3c), which is similar to other antimicrobial peptides, such as human β-defensin-3^51^ and the cathelicidin-related peptide LL-37^52^, suggesting that this region of GBP1 has a specific role in mediating bacteriolysis. Further, we stained *F. novicida* treated with GBP1 peptides or WLBU2 with SYTOX, followed by quantification using flow cytometry (Fig. 3d). We found that treatment of *F. novicida* with GBP1^28–67^ and WLBU2 induced a significant uptake of SYTOX, whereas treatment with other GBP1 peptides did not (Fig. 3d, Supplementary Fig. 9a). Importantly, we found that GBP1^28–67^ did not induce killing of *E. coli*, but treatment with WLBU2 led to death of the bacteria (Supplementary Fig. 9b), suggesting pathogen-selectivity of GBP1^28–67^.

The ability of GBP1^28–67^ to directly kill *F. novicida* suggests that it would require binding to the bacterial surface. To investigate this possibility, we incubated *F. novicida* or *E. coli* with FITC-tagged GBP1^28–67^ (called FITC-GBP1^28–67^) or a FITC-tagged control peptide and quantified their fluorescence signal (Fig. 3e–g). We also incubated these bacteria in the presence of SYTOX and observed that FITC-GBP1^28–67^ localized to and induced uptake of SYTOX in *F. novicida*, whereas this did not occur with *E. coli* (Fig. 3e–g). These data suggest that GBP1^28–67^ can directly bind to and induce the killing of *F. novicida* in a pathogen-selective manner.

To confirm whether charge and hydrophobicity are key features facilitating the antimicrobial activity of GBP1^28–67^, we labeled *F. novicida* with FITC-GBP1^28–67^ followed by exposure to (i) a higher concentration of NaCl which can affect interactions between peptides and membranes by altering charge and ionic strength, (ii) the non-ionic surfactant saponin which can alter hydrophobic interactions, or (iii) the anionic surfactant sarcosyl which can reduce both charge and hydrophobic interactions^53^. We found that *F. novicida* which had already been bound to FITC-GBP1^28–67^ and exposed to NaCl, saponin or sarcosyl resulted in a loss of FITC signal, whereas exposure to PBS did not (Fig. 3h). Exposure of *F. novicida* to NaCl, saponin or sarcosyl and subsequent removal of these solutions prior to the addition of FITC-GBP1^28–67^ did not impair the binding between FITC-GBP1^28–67^ and *F. novicida* (Supplementary Fig. 9c). These data suggest that a potential change in charge and hydrophobicity removes FITC-GBP1^28–67^ from *F. novicida*, rather than a loss of bacterial surface structures. Further, the reduction in FITC signal from *F. novicida* was not owing to quenching of the FITC signal by NaCl, saponin or sarcosyl, or the lysis of *F. novicida* by these reagents (Supplementary Fig. 9d, e). These data collectively suggest that a specific region of GBP1 mediates pathogen-selective binding and killing of bacteria.

To assess the antimicrobial kinetics of GBP1^28–67^, we incubated *F. novicida* with solvent control, GBP1^28–67^, or WLBU2 and then assessed bacterial viability at 0.5, 1, 2, 4, and 6 h time points. Compared to solvent control, GBP1^28–67^ had a robust antimicrobial effect against *F. novicida* from 2 h of incubation (Supplementary Fig. 9f). Furthermore, we also assessed the ability of GBP1^28–67^ to kill *F. novicida* in a range of physiological buffer conditions and confirmed its antimicrobial activity in PBS, saline and RPMI (Supplementary Fig. 9g). Many antimicrobial peptides and proteins, such as cathelicidin in immune cells and APOL3 in epithelial cells, are active in the cytoplasm^54,55^. Although GBP1^28–67^ can kill bacteria in PBS, saline, and RPMI, (Supplementary Fig. 9g), our in vitro antimicrobial assays do not fully recapitulate the complex cytosolic environment. Therefore, we cannot exclude the possibility that mGBP1 has no direct antimicrobial activity in the cytoplasm or that its intrinsic antimicrobial activity is not sufficient to trigger downstream signaling events without other host factors.

Previous studies have shown that GBP1 can target mammalian membranes^56,57^. To investigate whether GBP1 peptides disrupt mammalian membranes, we treated primary mouse BMDMs, African green monkey kidney cells Vero, human embryonic kidney cells HEK293, and human intestinal epithelial cells HT29 with GBP1^28–67^, GBP1^209–238^, GBP1^424–452^, and GBP1^558–577^. None of the peptides exhibited cytotoxicity against these cell types, quantified by SYTOX incorporation or lactate dehydrogenase (LDH) release (Supplementary Fig. 9h, i). These data suggest that a conserved N-terminal region of the globular head domain of GBP1 do not induce cytotoxicity in mammalian cells.

We next further examined the pathogen-selective potential exhibited by the GBP1^28–67^ peptide. To achieve this, we tested the killing activity of this peptide against a range of Gram-positive and Gram-negative bacteria (Supplementary Fig. 10a). We found that GBP1^28-67^ exhibited substantial antimicrobial activity against *F. novicida* and the Gram-negative pathogenic bacterium *N. meningitidis* (Supplementary Fig. 10a), but not against the Gram-negative bacteria *C. rodentium*, *E. coli*, *Pseudomonas aeruginosa*, *S. flexneri* and *S*. Typhimurium, or the Gram-positive bacteria *Bacillus cereus*, *L. monocytogenes*, and *Staphylococcus aureus* (Supplementary Fig. 10a).

We further confirmed that FITC-GBP1^28–67^ localized to and killed *N. meningitidis*, whereas this did not occur with the use of a FITC-control peptide (Supplementary Fig. 10b, c). Moreover, recombinant full-length mGBP1 also killed *N. meningitis* in a dose-dependent manner (Supplementary Fig. 10d). Immunofluorescence staining revealed that mGBP1 localized to *N. meningitidis*, resulting in the uptake of SYTOX (Supplementary Fig. 10e). Collectively, these data further suggest that this stretch of 40 amino acids within the head domain of GBP1 can selectivity bind and induce the killing of *F. novicida* and *N. meningitidis*.

We hypothesized that LOS of *N. meningitidis* might be a putative ligand of mGBP1, based on the finding that *Salmonella* LPS is a putative ligand of hGBP1^42–45^. To investigate this, we incubated recombinant full-length mGBP1 against WT *N. meningitidis* and an isogenic *lpxA* mutant of *N. meningitidis*, which lacks LOS^58^. We found that mGBP1 can kill both WT and Δ*lpxA* mutant strains (Supplementary Fig. 10f). These data indicate that, in the absence of LOS, mGBP1 can still bind and kill *N. meningitidis*, and suggest that more than one bacterial ligand may be recognized by mGBP1.

The stretch of amino acids that constitute GBP1^28–67^ are conserved across the mouse and human GBPs (Supplementary Fig. 11a). To determine whether the antimicrobial activity is conserved, we synthesized the equivalent region of GBP1^28–37^ within mouse GBP3 (GBP3^22–61^). Incubation of this peptide with *F. novicida* resulted in bacterial killing in a dose-dependent manner but not for *E. coli* (Supplementary Fig. 11b). Using SEM and transmission electron microscopy (TEM), we also observed bacterial membrane damage and bacteriolysis of *F. novicida* following incubation with GBP3^22–61^ (Supplementary Fig. 11c) confirming that the antimicrobial activity of this region is conserved in GBP3.

### The N-terminal alpha helix mediates GBP1 peptide killing

Given that a portion of the GBP1^28–67^ region overlaps with the GTPase domain, it is challenging to uncouple the GTPase activity from the antimicrobial activity of GBP1^28–67^ within the full-length protein. Therefore, we went on to narrow down the antimicrobial killing domain of GBP1^28–67^ and determine whether this region overlaps with any of the conserved motifs critical for GTPase activity. The killing domain of GBP1^28–67^ is either surface exposed or buried within the tertiary structure of GBP1. Surface modeling revealed that the two flanking alpha helices, GBP1^28–38^ and GBP1^46–67^, are surface-exposed, whereas the middle beta-sheet, GBP1^39–45^ is hidden (Supplementary Fig. 11d). To determine which of these regions contributed to antimicrobial activity, we synthesized the peptides GBP1^28–38^ and GBP1^46–67^ which correspond to N-terminal and C-terminal flanking alpha helices, respectively. We were unable to generate the middle beta-sheet, GBP1^39–45^, due to its high hydrophobicity, however, the antimicrobial activity of this region was determined using GBP1^38–67^ (middle beta-sheet + C-terminal flanking alpha helix). We found that GBP1^28–38^ killed *F. novicida* and *N. meningitidis* similar to that observed for GBP1^28–67^ (Supplementary Fig. 11e). Importantly, GBP1^38–67^ and GBP1^46–67^ had little or no antimicrobial activity (Supplementary Fig. 11e). GBP1^28–38^ was unable to kill *E. coli*, confirming its antimicrobial specificity (Supplementary Fig. 11e). Based on these data, we narrowed the antimicrobial N-terminal region of mGBP1 down to 11 amino acids. Importantly, the sequence of GBP1^28–38^ does not contain the GTP binding motifs or residues essential for the GTPase activity^59^.

### GBP1 ruptures the bacterial membrane leading to DNA release

**Fig. 4:**
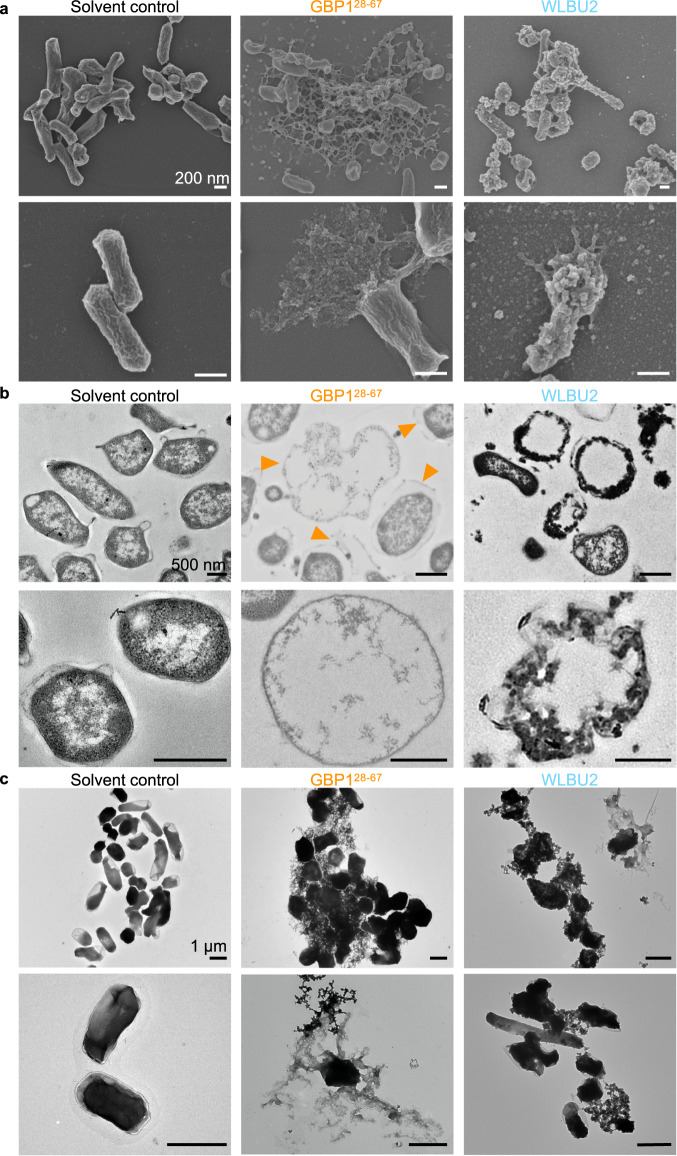
GBP1 peptide induces membrane disruption, expulsion of cytoplasmic and membranous content from *F. novicida*. **a** Scanning electron microscopy and (**b**) transmission electron microscopy analysis of the morphology of *F. novicida* 12 h after treatment with solvent control, 100 μg/mL of GBP1^28–67^ or WLBU2. **c** Negative-stain transmission electron microscopy analysis of *F. novicida* 12 h after treatment with solvent control, 100 μg/mL of GBP1^28–67^ or WLBU2. Scale bars, 200 nm (**a**), 500 nm (**b**) and 1 μm (**c**). Orange arrow heads indicate bacteria with disrupted cell membrane. Data are representative of three independent experiments (**a**–**c**).

Antimicrobial peptides or proteins can induce killing of bacteria via several mechanisms, including pore formation, membrane thinning or thickening, non-lytic membrane depolarization, oxidization of lipid components, or translocation of peptides targeting essential biological processes within the bacteria^60^. We used SEM and TEM to investigate the membrane integrity and ultrastructure of *F. novicida* following treatment with GBP1^28–67^ (Fig. 4a–c). We observed disruption of the membrane integrity, membrane rupture, and expulsion of intracellular content in bacteria treated with GBP1^28–67^ (Fig. 4a, b). The characteristics of cell death induced by GBP1^28–67^ were distinct from those of WLBU2, which exhibited apoptosis-like blebbing and apoptosis-like bodies (Fig. 4a, b). Under transmission electron microscopy coupled with negative staining to detect nucleic acid and proteinaceous material, we observed cytoplasmic expulsion of *F. novicida* following killing by GBP1^28–67^ (Fig. 4c). To confirm the release of bacterial DNA following treatment with GBP1^28–67^, we stained *F. novicida* with a DNA dye, Hoechst 33342, followed by treatment with GBP1^28–67^ or a solvent control and quantification of the release of the DNA dye by spectrometry (Supplementary Fig. 11f). We observed a substantial release of DNA from *F. novicida* following the addition of GBP1^28–67^, but not of the control peptide (Supplementary Fig. 11f). Indeed, GBP1^28–67^ did not induce the release of DNA from *E. coli* (Supplementary Fig. 11f). These data support the observation that a small region of GBP1 is sufficient to induce killing of certain bacteria, leading to expulsion of cytoplasmic content containing DNA, which is important for inflammasome sensing.

### GBPs are not required for the cytosolic entry of bacteria

The ability of certain GBPs to directly kill *F. novicida* as a mechanism to control bacterial replication does not exclude the possibility that these proteins can prevent entry of the bacteria into the cytoplasm of host cells. Cytosolic pathogens, such as *F. novicida*, escape from the pathogen-containing vacuole into the cytoplasm of host cells. This biological process allows *F. novicida* to be used as a vehicle to deliver ligands, such as *E. coli* LPS, into the cytoplasm of a cell and to interrogate the role of GBPs in mediating cytosolic escape of *F. novicida* (Supplementary Fig. 12a)^38^. We took advantage of this cytosolic delivery technique and infected WT, *Gbp1*^–/–^, *Gbp2*^–/–^, *Gbp3*^–/–^, *Gbp5*^–/–^, *Casp11*^–/–^, *Aim2*^–/–^, and *Aim2*^–/–^*Nlrp3*^–/–^ BMDMs with *F. novicida* in the presence or absence of ultrapure LPS from *E. coli* to investigate whether *E. coli* LPS can be efficiently introduced into the cytoplasm to activate the inflammasome in the absence of GBPs. In the absence of *E. coli* LPS, *F. novicida*-induced cleavage of caspase-1 and GSDMD and secretion of IL-1β, IL-18 and LDH required GBP1, GBP2, GBP3, GBP5, and AIM2, but not caspase-11 (Supplementary Fig. 12b, c). Importantly, we found that WT, *Gbp1*^–/–^, *Gbp2*^–/–^, *Gbp3*^–/–^, *Gbp5*^–/–^, and *Aim2*^–/–^ BMDMs all released similar levels of IL-1β, IL-18 and LDH in response to infection with *F. novicida* in the presence of *E. coli* LPS (Supplementary Fig. 12c). These results demonstrate successful delivery of *E. coli* LPS into the cytoplasm by *F. novicida* and activation of the LPS-sensing caspase-11-NLRP3 inflammasome even in the absence of GBPs. Indeed, *Aim2*^–/–^*Nlrp3*^–/–^ BMDMs infected with *F. novicida* in the presence of *E. coli* LPS did not result in caspase-1 or GSDMD cleavage, nor the release IL-1β, IL-18, and LDH (Supplementary Fig. 12b, c). The level of TNF was similar across all genotypes of BMDMs, suggesting that the ‘priming’ signal is intact in these cells (Supplementary Fig. 12c). These results further validate that GBPs do not interfere with the ability of *F. novicida* to escape the vacuole into the cytoplasm and that GBPs directly target the bacteria within the cytoplasm.

### GBPs provide host protection against bacterial infection

**Fig. 5:**
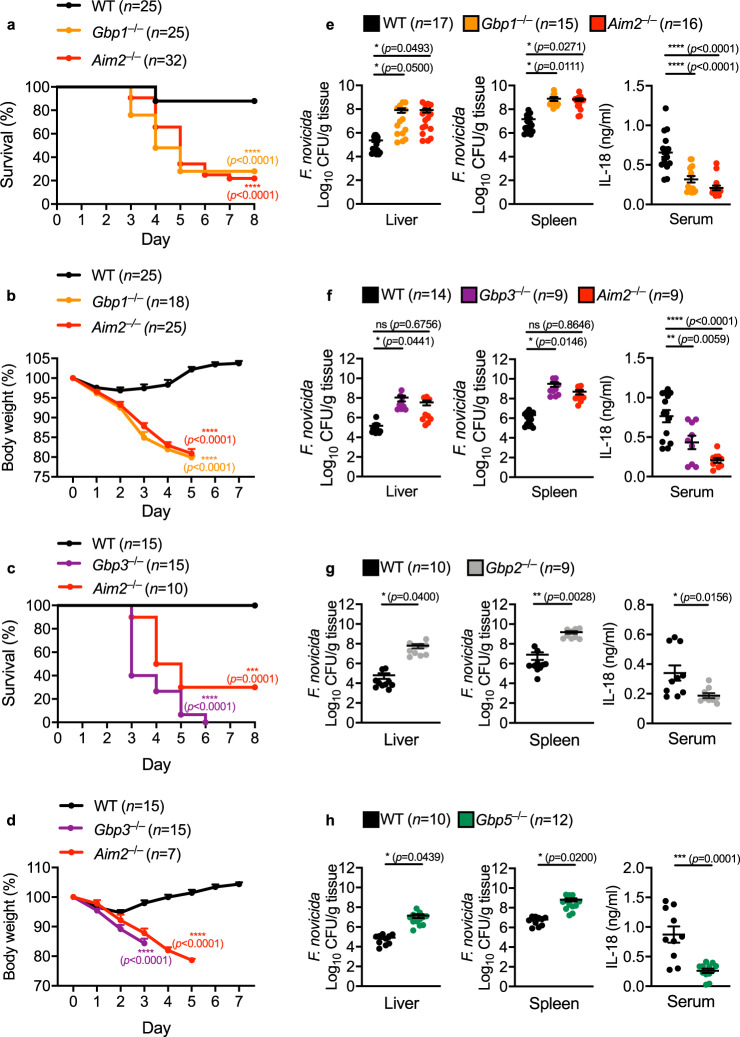
GBP1, GBP2, GBP3 and GBP5 provide host protection against *F. novicida* infection in vivo. **a** Survival of 7-week-old WT mice (*n* = 25), *Gbp1*^–/–^ mice (*n* = 25) and *Aim2*^–/–^ mice (*n* = 32) infected subcutaneously with 1.2 × 10^6^ colony-forming units (CFUs) of *F. novicida*. **b** Body weight of 7-week-old WT mice (*n* = 25), *Gbp1*^–/–^ mice (*n* = 18) and *Aim2*^–/–^ mice (*n* = 25) 0–7 d after subcutaneous infection with 1.2 × 10^6^ CFUs of *F. novicida*, presented relative to initial body weight at day 0, set as 100%. **c** Survival of 7-week-old WT mice (*n* = 15), *Gbp3*^–/–^ mice (*n* = 15) and *Aim2*^–/–^ mice (*n* = 10) infected subcutaneously with 1.2 × 10^6^ colony-forming units (CFUs) of *F. novicida*. **d** Body weight of 7-week-old WT mice (*n* = 15), *Gbp3*^–/–^ mice (*n* = 15) and *Aim2*^–/–^ mice (*n* = 7) 0–7 d after subcutaneous infection with 1.2 × 10^6^ CFUs of *F. novicida*, presented relative to initial body weight at day 0, set as 100%. **e** Bacterial burden in the liver (left) and spleen (middle) and concentration of IL-18 in the serum (right) of 7-week-old WT mice (*n* = 17), *Gbp1*^–/–^ mice (*n* = 15) and *Aim2*^–/–^ mice (*n* = 16) on day 3 after infection with 6 × 10^5^ CFUs of *F. novicida*. **f** Bacterial burden in the liver (left) and spleen (middle) and concentration of IL-18 in the serum (right) of 7-week-old WT mice (*n* = 14), *Gbp3*^–/–^ mice (*n* = 9) and *Aim2*^–/–^ mice (*n* = 9) on day 3 after infection with 6 × 10^5^ CFUs of *F. novicida*. **g** Bacterial burden in the liver (left) and spleen (middle) and concentration of IL-18 in the serum (right) of 7-week-old WT mice (*n* = 10) and *Gbp2*^–/–^ mice (*n* = 9) on day 3 after infection with 6 × 10^5^ CFUs of *F. novicida*. **h** Bacterial burden in the liver (left) and spleen (middle) and concentration of IL-18 in the serum (right) of 7-week-old WT mice (*n* = 10) and *Gbp5*^–/–^ mice (*n* = 12) on day 3 after infection with 6 × 10^5^ CFUs of *F. novicida*. Each symbol represents an individual mouse (**e**–**h**). ns no statistical significance; *P < 0.05, **P < 0.01; ***P < 0.001, ****P < 0.0001 (log-rank test (**a** and **c**) or one-way ANOVA with Dunnett’s multiple-comparisons test (**e**, **f**, mean and s.e.m. in **e,** **f**)) or two-tailed *t*-test (**b**, **d**, **g**, **h**, mean and s.e.m. in **b**, **d**, **g**, **h**). Data are pooled from two or three independent experiments (**a**–**h**). Source data are provided as a Source data file.

Given that GBPs facilitated activation of the inflammasome in primary macrophages, we speculated that GBPs would offer protection against *F. novicida* in a mouse model of infection. To this end, we infected WT, *Gbp1*^–/–^, *Gbp2*^–/–^, *Gbp3*^–/–^, *Gbp5*^–/–^, and *Aim2*^–/–^ mice with *F. novicida* and monitored their susceptibility to infection. *Gbp1*^–/–^ mice lost more body weight compared with WT mice and 72% of *Gbp1*^–/–^ mice succumbed to infection within 7 days, whereas 88% of the WT mice survived (Fig. 5a, b). Similarly, *Gbp3*^–/–^ mice (100%) lost more body weight compared with WT mice and succumbed to infection within 7 days, whereas all WT mice survived (Fig. 5c, d). In addition, *Gbp1*^–/–^ and *Gbp3*^–/–^ mice harbored significantly more viable *F. novicida* in the liver and spleen compared with WT mice (Fig. 5e, f). Analysis of serum IL-18 showed that *Gbp1*^–/–^ and *Gbp3*^–/–^ mice had an impaired ability to produce this inflammasome-dependent cytokine following infection with *F. novicida* (Fig. 5e, f). Reduced level of serum IL-18 was also observed in *Aim2*^–/–^ mice (Fig. 5e, f). In addition, we observed substantial increased levels of viable *F*. novicida in the liver and spleen of *Gbp2*^–/–^ and *Gbp5*^–/–^ mice and reduced serum IL-18 in these mice (Fig. 5g, h), consistent with a previous study^37^. Together, these results highlighted a crucial role for individual GBPs in the host defense against *F. novicida* infection, through a mechanism dependent on inflammasome signaling.

## Discussion

GBP recruitment to intracellular pathogens is critical for host defense and innate immune responses. Previous studies have used *Gbp*^chr3^-KO cells to demonstrate the role of mouse GBPs in restricting intracellular bacterial proliferation and promoting inflammasome activation^61^. In this study, we investigated the contribution of individual GBPs encoded on chromosome 3. In mice, GBP2 and GBP5 have been shown to facilitate AIM2 inflammasome activation in response to *F. novicida* infection^36,37^. We further show that GBP1 and GBP3 can also promote AIM2 inflammasome activation induced by *F. novicida* infection. Why at least four different GBPs are required to promote this host response is unclear. Importantly, we show that in *Gbp2*^–/–^, *Gbp3*^–/–^, and *Gbp5*^–/–^ BMDMs, *F. novicida* remained coated by GBP1 but had reduced ability to induce AIM2 inflammasome activation. We reasoned that although GBP1 targeted *F. novicida* in *Gbp2*^–/–^, *Gbp3*^–/–^, and *Gbp5*^–/–^ BMDMs, recruitment of AIM2 may require additional host molecules in order to trigger more downstream events, such as inflammasome activation. However, we cannot exclude that, in BMDMs, GBP1 is devoid of antimicrobial activity in the absence of GBP2, GBP3, or GBP5. Indeed, previous work by us has shown that IRGB10 recruitment is required to trigger *F. novicida* bacteriolysis and AIM2 inflammasome activation^38^. However, we cannot exclude the possibility that the reduced AIM2 inflammasome activation observed in *Gbp1*^–/–^ and *Gbp3*^–/–^ BMDMs following *F. novicida* infection is due to a lack of IRGB10 recruitment instead of intrinsic antimicrobial activity of GBP1 and GBP3. Although we show an increased bacterial burden in BMDMs deficient in GBP1 and GBP3, suggesting that they have a role in mediating bacterial killing, we could not directly observe GBP1- and GBP3-mediated bacteriolysis in BMDMs due to technical difficulties. Therefore, our conclusions regarding GBP1- and GBP3-mediated bacteriolysis are based on AIM2 activation (bacterial DNA release in the cytoplasm suggestive of bacteriolysis) and potentially reduced cell death observed in GBP-deficient BMDMs affecting the intracellular bacterial burden in these cells.

Human GBP1 can initiate the assembly of a multi-GBP complex, including human GBP3, such that this GBP complex activates caspase-4^43–45^. However, in mice, we show that mGBP1 is not required for the recruitment of mGBP2, mGBP3, or mGBP5 to intracellular *F. novicida*. Therefore, our data highlight that differences between human GBPs and mouse GBPs exist in the context of cell-autonomous immunity and inflammasome activation. Further investigations might consider how different recruitment strategies to intracellular pathogens might affect pathogen killing and activation of inflammasomes.

We identified a pathogen-selective peptide that binds to and mediates killing of *F. novicida* and *N. meningitis*, but not other bacteria or mammalian cells. We observed that the killing domain (amino acid position 28–37 in mouse GBP1) is a highly conserved region across human and murine GBPs. Indeed, this highly conserved region may in part explain why mouse GBP1, GBP2, GBP3, and GBP5 all restrict intracellular *F. novicida* proliferation. While we focused on the antimicrobial activity of mGBP1 and its N-terminal α-helix (amino acids 28–38) of mGBP1, the antimicrobial functions of endogenous mGBP1 during *F. novicida* infection likely require multiple features, including its GTPase activity and the CaaX box. Indeed, previous studies have shown that both these features are important for hGBP1 to target membrane structures^62^. A possible scenario is that these domains facilitate the delivery of GBPs to cytosolic bacteria, subsequently enabling the N-terminal α-helix (amino acids 28–38) to induce bacterial rupture. Additionally, the human GBP1^K61-K63^ has also been shown to directly bind to LPS from Gram-negative bacteria via electrostatic forces^43^. However, these three consecutive lysine residues are not found in mouse GBPs encoded on chromosome 3, yet remarkably, mouse GBPs can still be recruited to the surface of Gram-negative bacteria. Collectively, these findings suggest that human and murine GBPs have different biological activities and may use different regions and/or mechanisms to bind to bacteria.

Human GBP1 can bind to bacterial LPS, suggesting that GBPs might represent a class of cytosolic sensors^42–44,63^. Our observations that mouse GBP1 mediates the inflammasome response to a subset of Gram-negative bacteria suggest that other bacterial ligands can also be recognized by GBP1. Indeed, both human and mouse GBP1 can be recruited to pathogen-containing vacuoles encasing the parasite *T. gondii*, a pathogen that lacks LPS^40,41,56,57^, indicating that the repertoire of microbial ligands that GBP1 can bind to expands beyond the bacterial domain. Moreover, we also found that mGBP1 can induce killing of *N. meningitis* and its mutant which lacks LOS, indicating that additional bacterial ligands can also interact with mGBP1. We speculate that the selectivity of GBP1 towards *F. novicida* and *N. meningitidis* is mediated by the presence of unique ligands present within these bacteria.

An exciting prospect in medicine is the possibility to harness the mammalian immune system as a source of disease-fighting antimicrobial proteins. The identification of antimicrobial peptides inspired by the inflammasome pathways might be relevant in the fight against antimicrobial resistance. Indeed, inappropriate use of antibiotics has led to a rise in the prevalence of antibiotic-resistant bacteria worldwide. The study of the mammalian immune system can inform the design and development of more selective and effective antimicrobials.

## Methods

### Mice

C57BL/66NcrlAnu mice and *Mefv*^–/–^ mice were sourced from The Australian National University. *Nlrp3*^–/–^^64^ and *Casp11*^–/–^^65^ mice were sourced from The Jackson Laboratory. *Nlrc4*^–/–^ mice^66^ were sourced from the University of Queensland. *Aim2*^–/–^ mice^31^ were sourced from Genentech. *Gbp*^chr3^-KO mice^49^ were sourced from Osaka University. *Aim2*^–/–^ *Nlrp3*^–/–^ mice were generated by crossing *Aim2*^–/–^ mice and *Nlrp3*^–/–^ mice. All mice are on the C57BL/66NcrlAnu background, or backcrossed to C57BL/66NcrlAnu background for at least 10 generations.

Mice with a genomic deletion of either GBP1, GBP2, GBP3, GBP5, or GBP7 (called *Gbp1*^–/–^, *Gbp2*^–/–^, *Gbp3*^–/–^, *Gbp5*^–/–^, and *Gbp7*^–/–^ mice respectively) were generated by Cas9/CRISPR-mediated genome editing technology^67,68^. The mouse genomic sequences were obtained from Ensembl (Ensembl.org). Cas9 protein (Cat#:1081059) and the single guide RNA (sgRNA) were purchased from IDT (Singapore) with the following sequences: *Gbp1* sgRNA1 5’- AGACAACTCAGCTAACTTTG TGG-3’ targeting exon 5 resulting in a 2 bp deletion. *Gbp2* sgRNA1 5’-GTGTGTGCCTCACCCCAAGA AGG-3’ *Gbp2* sgRNA2 5’-GACGATTCCGCTAACTTTGT GGG-3’ and *Gbp2* sgRNA3 5’-TCGTTGCTCAGACTTGCTGG AGG-3’ targeting exons 3, 5 and 8 respectively resulting in a 6,669 bp deletion. *Gbp3* sgRNA1 5’-ATTGTTGGTTTATATCGTAC AGG-3’, and *Gbp3* sgRNA2 5’-GGCAAAATCGAGCCCCAGAG AGG-3’ targeting exons 2 and 5 respectively resulting in a 3,404 bp deletion. *Gbp5* sgRNA1 5’- ATTGTGGGTCTTTATCGCAC AGG-3’, *Gbp5* sgRNA2 5’- CTCAAACATTCAATCTACCG CGG-3’ and *Gbp5* sgRNA3 5’-CTGCCCGGCTCGAAGCACAG AGG-3’ targeting exons 2, 6, and 10 respectively resulting in a 7,268 bp deletion. *Gbp7* sgRNA1 5’-GAGGATCACTCAGCCTGTAG TGG-3’ and *Gbp7* sgRNA3 5’-CTGAGGGAGAGCATCTCACG TGG-3’ targeting exons 2 and 8 respectively.

The nucleases were delivered into the pronucleus of the C57BL/6NCrl fertilized zygotes at the following concentrations: Cas9 protein (50 ng/µL) was co injected with a mixture of sgRNA (2.5 ng/µL). After the micro-injection of the zygotes were incubated overnight at 37 °C under 5% CO_2_ and two-cell stage embryos were surgically transferred into the ampulla of the pseudopregnant CFW/Crl mice. DNA was extracted from the ear punches of the mice using a crude DNA extraction protocol and PCR amplification. The PCR products were then purified with a PCR Clean-Up System (Promega) kit according to the manufacturer’s instructions. Sanger sequencing was performed in the Biomolecular Resource facilities at the Australian National University to identify a 2 bp deletion in exon 2 of *Gbp1*, a 6669 bp deletion between exon 3 and 6 of *Gbp2*, a 3404 bp deletion between exon 2 and 5 of *Gbp3*, a 7268 bp deletion between exon 2 and 10 of *Gbp5*, a 7 bp deletion in exon 2 and 1 bp deletion in exon 8 of *Gbp7*.

Male and female mice of 6–8 weeks old were used. Mice were bred and maintained at The Australian National University under specific pathogen-free conditions. All animal studies were conducted in accordance with the Protocol Number A2020/19 approved by The Australian National University Animal Experimentation Ethics Committee.

### Microbial culture

*F. novicida* strain EXO186 (Queensland Health Forensic and Scientific Services) was grown in BBL Trypticase Soy Broth (TSB) (211768, BD) supplemented with 0.2% L-cysteine (BP376-100, Thermo Fisher Scientific) overnight under aerobic conditions at 37 ^o^C. *Citrobacter rodentium* (51459, American Type Culture Collection), *Escherichia coli* (1175, American Type Culture Collection), *Salmonella* Typhimurium SL1344, *Staphylococcus aureus* F-182 (43300, American Type Culture Collection), *Shigella flexneri* 2457 T (700930, American Type Culture Collection), *Pseudomonas aeruginosa* CLBU 20 PAK (University of Technology Sydney) and *Bacillus cereus* (14579, American Type Culture Collection) were grown in Luria-Bertani (LB) media (244620, BD) overnight under aerobic conditions 37 ^o^C. *Listeria monocytogenes* 53XXIII (15313, American Type Culture Collection), *Neisseria meningitidis* (10036, DSMZ-German Collection of Microorganisms and Cell Cultures), *Neisseria meningitidis* (FAM20, Stockholm University) and *Neisseria meningitidis ΔIpxA* (FAM20, Stockholm University)^58^ were grown in brain heart infusion (BHI) media (211059, BD) overnight under aerobic conditions 37^o^C and then subcultured (1:10) into fresh BHI followed by 3 h incubation. Overnight cultures were either used directly or subcultured (1:10) into fresh media and grown for 3 h to generate log-phase culture. A clinical isolate of *Clostridium difficile* positive for TcdA and TcdB toxin (ACT Pathology) was grown BHI media for 48 h under anaerobic conditions at 37 ^o^C. The toxin-containing supernatant was then harvested by centrifugation. The MCMV Smith MSGV strain (VR-1399, American Type Culture Collection) was propagated and expanded in M2-10B4 murine bone marrow stromal cells (CRL-1972, American Type Culture Collection) and concentrated by ultracentrifugation.

### Bone marrow-derived macrophages

Primary BMDMs were cultured for 5–6 days in Dulbecco’s Modified Eagle Medium (DMEM) (11995073, Gibco Thermo Fisher Scientific) supplemented with 10% fetal bovine serum (FBS; F8192, Sigma), 30% L929-conditioned media and 1% penicillin and streptomycin (10378016, Gibco Thermo Fisher Scientific) as previously described^38^. BMDMs were seeded in antibiotic-free media at a concentration of 1 × 10^6^ cells per well in 12-well plates.

For activation of the AIM2 inflammasome, BMDMs were infected with *F. novicida* (MOI 100, 10–16 h), *L. monocytogenes* (MOI 100, 20 h), MCMV (MOI 10, 10 h) or transfected with poly(dA:dT) (tlrl-patn, InvivoGen) or plasmid pcDNA3.1 DNA (V79020, Thermo Fisher Scientific). For DNA transfection, each reaction consisted of 5 µg of poly(dA:dT) or plasmid DNA resuspended in PBS and mixed with 0.3 µl of Xfect polymer in Xfect reaction buffer (631318, Clontech Laboratories, Inc.). After 20 min, DNA complexes were added to BMDMs in Opti-MEM (31985-070, Thermo Fisher Scientific) and incubated for 4 h. For activation of the NLRC4 inflammasome, BMDMs were infected with *S*. Typhimurium (MOI 2, 4 h) or *Pseudomonas aeruginosa* (MOI 5, 4 h). For activation of the canonical NLRP3 inflammasome, BMDMs were infected with *B. cereus* (MOI 5, 3–6 h), *Staphylococcus aureus* (MOI 25, 20 h) or primed using 500 ng/mL ultrapure LPS from *E. coli* (ALX-581-014-L002, Enzo Life Sciences) for 3 h and stimulated with 5 mM ATP (10127531001, Roche) or 10 µM nigericin (N7143, Sigma) for 4 h. For activation of the non-canonical NLRP3 inflammasome, BMDMs were infected with *C. rodentium* (MOI 20, 20 h), *E. coli* (MOI 25, 20 h) or transfected with *E. coli* LPS (5 µg) using Xfect as described above. Gentamicin (50 µg/mL, 15750-060, Thermo Fisher Scientific) was added after 4–6 h (*C. rodentium*, *E. coli*) post-infection to kill extracellular bacteria. For activation of the Pyrin inflammasome, BMDMs were primed with LPS for 3 h and stimulated with 100 µL of *C. difficile* culture supernatant for 20 h.

BMDMs were infected with *F. novicida* (MOI 100, 4 h) for qRT-PCR analyses of *Il1b*, *Il18*, *Il6*, *Cxcl1*, *Tnf*, *Ifnb, Gbp1, Gbp2, Gbp3, Gbp5*, and *Gbp7* expression or ELISA for IFN-β; *F. novicida* (MOI 100, 10–16 h) for ELISA of IL-1β, IL-18, IL-6, KC and TNF; *F. novicida* (MOI 100, 5-60 min) for pIkB, IkB, pERK, ERK expression; *F. novicida* (MOI 25, 0–20 h) for GBP expression; *F. novicida* (MOI 25, 8 h) for immunofluorescence staining of GBPs followed by 1 h of incubation in gentamicin (50 µg/mL) containing media; *F. novicida* (MOI 100, 10–16 h) with or without LPS for the cytosolic escape assay. For CFU analysis, supernatant from BMDMs infected with *F. novicida* for 3, 7, and 11 h was replaced with media containing 50 μg/mL gentamicin (Gibco). Cells were incubated for an additional 1 h, washed three times with PBS, lysed in water and scraped from plates. The intracellular bacteria were serially diluted before plating onto TSB agar.

### LA-4 mouse lung epithelial cells

Mouse lung epithelial cells, LA-4, expressing FLAG-tagged OVA, mGBP1, mGBP2, mGBP3, mGBP5 or mGBP7 were cultured in Ham’s F-12K (Kaighn’s) Medium (21127022, Gibco Thermo Fisher Scientific) supplemented with 10% fetal bovine serum and 1% penicillin and streptomycin. LA-4 cells were seeded at a concentration of 1 × 10^5^ cells per well in 12-well plates. To induce the expression of FLAG-tagged GBPs, LA-4 cells were primed with 10 µg/mL of doxycycline hyclate (D9891, Merck) for 48 h. LA-4 cells were left untreated or treated with 100 U/mL of mouse IFN-γ (130-105-790, Miltenyi Biotec) for 24 h before they were infected with *F. novicida* (MOI 100, 16 h) followed by 1 h incubation in gentamicin (50 µg/mL). For immunofluorescence staining of GBPs, LA-4 cells were washed three times with PBS before fixed in 4% paraformaldehyde.

To overexpress IRGB10, LA-4 cells were transfected with plasmid encoding dTomato-IRGB10 (1 µg/mL) using Xfect polymer in Xfect reaction buffer (631318, Clontech Laboratories, Inc.) for 4 h prior to bacterial infection.

### Lactate dehydrogenase assay

Levels of lactate dehydrogenase released by cells were determined using the CytoTox 96 Non-Radioactive Cytotoxicity Assay according to the manufacturer’s instructions (G1780, Promega).

### IncuCyte and cytotoxicity analysis

To track cell viability in real time, BMDMs were stimulated in presence of the SYTOX Green nuclear stain that penetrates compromised membranes (1 μM; S7020; Life Technologies). Cell death was monitored over 22 h using the IncuCyte Zoom imaging system (Essen Biosciences) and data was collected using IncuCyte v2018B.

### Cytokine analysis

Cytokine concentrations from BMDMs were calculated using a multiplex ELISA IL-1β, TNF, KC and IL-6 (MCYTOMAG-70K, EMD Millipore), an IFN-β ELISA (MECY2MAG-73K, EMD Millipore) or an IL-18 ELISA (BMS618-3TEN, Thermo Fisher) according to the manufacturers’ instructions. Cytokines were quantified on a MAGPIX (Luminex) analyzer and data was collected using xPONENT v4.2.

### Real Time qRT-PCR analysis

RNA was extracted from BMDMs using TRIzol (15596018, Thermo Fisher Scientific). Isolated RNA was converted into cDNA using the High-Capacity cDNA Reverse Transcription Kit (4368814, Thermo Fisher Scientific). RT-PCR was performed and analyzed on a QuantStudio 12 K Flex Real-Time PCR System (Applied BioSystems) with PowerUp SYBR Green Mastermix (A25741, Thermo Fisher Scientific). Primer sequences can be found in Supplementary Table 6.

### Immunoblotting analysis

For caspase-1 and GSDMD immunoblotting, BMDMs and supernatant were lysed in lysis buffer and sample loading buffer containing sodium dodecyl sulfate (SDS) and 100 mM dithiothreitol (DTT). For immunoblotting of GBPs, Pyrin, pIκBα, IκBα, pERK, ERK, and β-actin, the supernatant was removed and BMDMs were washed once with PBS, followed by lysis in radio-immunoprecipitation buffer and sample loading buffer containing SDS and 100 mM DTT. Proteins were separated on 8–12% polyacrylamide gels. Following electrophoretic transfer of proteins onto polyvinyldifluoride (PVDF) membranes (IPVH00010, Millipore), membranes were blocked in 5% skim milk in tris-buffered saline with Tween-20 (TBST) and incubated overnight with primary antibodies against caspase-1 (1:3000 dilution, AG-20B-0042, Adipogen), GSDMD (1:3000 dilution, ab209845, Abcam), GBP1 (1:20 dilution^69^), GBP2 (1:1000 dilution, CAC07820, Biomatik), GBP3 (1:1000 dilution, SA0035 RB1060, Biomatik), GBP5 (1:1000^70^), GBP7 (1:1000 dilution, SA0039 RB1065, Biomatik), Pyrin (1:100 dilution, 195975, Abcam), pIkB (1:1000 dilution, 2859, Cell Signaling Technologies), IkB (1:1000 dilution, 9242, Cell Signaling Technologies), pERK (1:1000 dilution, 9101, Cell Signaling Technologies), ERK (1:1000 dilution, 9102, Cell Signaling Technologies), β-actin (1:10,000 dilution, 8457, Cell Signaling Technologies). PVDF membranes were then incubated with anti-rabbit (1:5000 dilution, 111035144, Jackson ImmunoResearch) or anti-mouse (1:5000 dilution, 115035146, Jackson ImmunoResearch) horseradish peroxidase-conjugated secondary antibodies for 1 h and proteins were visualized using Clarity Western ECL Substrate (170-5061, BioRad) and the ChemiDoc Touch Imaging System (BioRad). Immunoblots were analyzed using ImageLab Software v6.01

### Cloning

For recombinant protein expression, the DNA sequence of mGBP1 (CCDS: 38658.1 [https://www.ncbi.nlm.nih.gov/CCDS/CcdsBrowse.cgi?REQUEST = CCDS&DATA = 38658.1&ORGANISM = 0&BUILDS = CURRENTBUILDS]) was synthesized by Genscript and cloned into pET28a(+)-TEV between NdeI and XhoI restriction sites, thereby creating a 6x-His Tag at the N-terminus of mGBP1.

For overexpression studies, the DNA sequence of IRGB10 (ENA: ABF85830.1 [https://www.ebi.ac.uk/ena/browser/view/ABF85830.1]) fused to dTomato at the N-terminus was synthesized by Genscript and cloned into pCDNA3.1(+)-myc-HisA between EcoRI and XhoI restriction sites.

### Recombinant protein expression and purification

The BL21(DE3) *E. coli* strain (C2527H, NEB) was transformed with pET-28a(+)-TEV plasmid containing the sequence for mGBP1 and transformants were selected with 50 µg/mL kanamycin (10106801001, Roche). A single colony was used to inoculate a starter culture of 10 mL LB_Kan_ broth (LB broth + 50 μg/mL kanamycin) which was incubated at 37 °C, shaking (180 rpm) overnight. The overnight culture was diluted 1:100 into 800 m L of LB_Kan_ broth and incubated at 37 °C, shaking (180 rpm) for 2–3 h until an OD_600_ of 0.7 was obtained. Cultures were cooled to room temperature, expression was induced by adding isopropyl β-D-1-thiogalactopyranoside (0.5 mM; IPTG, Roche) and the incubation continued at 18 °C with shaking (180 rpm) overnight. The culture was centrifuged (5000 × *g*, 20 min, 4 °C) to pellet the bacteria and stored at −80 °C until required. The cell pellet was resuspended in lysis buffer (50 mM NaH_2_PO_4_, 300 mM NaCl, 10 mM imidazole, 5% glycerol (v/v), 5 mM MgCl_2_, 0.01% Triton X-100, pH 8.0) supplemented with lysozyme (250 µg/mL), Benzonase nuclease (50 U/mL) and protease inhibitor cocktail (11697498001, Roche) and incubated with gentle agitation at 4 °C for 1 h. Cells were subsequently disrupted by sonication and centrifuged (18,000 × *g*, 30 min, 4 °C) to pellet cellular debris. The supernatant was passed through a 0.22 µm filter (SLGP033RS, Merck) and mGBP1 was purified using Ni-NTA agarose resin (30210, Qiagen) as per the manufacturers’ instructions. The purity of eluted proteins was analyzed by SDS-PAGE and Coomassie blue staining. Purified proteins were dialyzed in DPBS (14190, ThermoFisher) containing 20 mM Tris and 20% glycerol (v/v), pH 7.5.

### Immunofluorescence staining

To visualize ASC speck formation, untreated or treated BMDMs were washed three times with PBS and fixed with 4% paraformaldehyde at room temperature for 15 min, followed by blocking in 10% normal goat serum (005000121, Jackson ImmunoResearch) supplemented with 0.1% saponin (47036, Sigma) for 1 h. Cells were incubated with a rabbit anti-ASC antibody (1:500 dilution, clone AL177, AG-25B-0006-C100, AdipoGen) overnight at 4 °C. An anti-rabbit secondary Rhodamine red antibody (1:500 dilution, 111295144, Jackson ImmunoResearch) was used. Cells were counterstained in 4,6-diamidino-2-phenylindole (DAPI) mounting medium (H-1200, Vecta Labs) or ProLong Gold with DAPI (P36941, Thermo Fisher Scientific). Inflammasome specks and BMDMs were visualized, counted and imaged using a Zeiss Axio Observer.

For GBP staining, BMDMs were infected for the indicated times and washed three times with PBS. Cells were fixed and blocked as described above and stained using either rabbit anti-GBP1 (1:20 dilution^69^), rabbit anti-GBP2 (1:500 dilution^70^), rabbit anti-GBP3 (1:20 dilution, SA0035 RB1059, Biomatik) rabbit anti-GBP5 (1:200 dilution^70^), or rabbit anti-GBP7 (1:200 dilution^70^), overnight at 4**°**C. An anti-rabbit secondary Rhodamine red antibody (1:500 dilution, 111295144, Jackson ImmunoResearch) was used. Cells were counterstained in DAPI mounting medium (Vecta Labs). GBPs were visualized and imaged using a Zeiss Axio Observer.

Intracellular *F. novicida* were stained with anti-*F. novicida* primary antibody (1:500 dilution^71^), overnight at 4 **°**C. The secondary antibody used was an Alexa Fluor 488 anti-chicken IgG (1:500 dilution, 103545155, Jackson ImmunoResearch). *F. novicida* were visualized and imaged using a Zeiss Axio Observer and quantified manually. For co-staining with GBPs, BMDMs were simultaneously stained with the anti-*F. novicida* antibody as above and either anti-GBP1, anti-GBP2, anti-GBP3 or anti-GBP5; LA-4 cells were stained with the anti-*F. novicida* antibody as above and anti-DYKDDDDK (1:200 dilution, 2368 S, Cell Signaling Technology). Cells were washed five times with PBS. An Alexa Fluor 488 anti-chicken IgG (1:500 dilution, 103545155, Jackson ImmunoResearch) and Alexa Fluor 647 anti-rabbit IgG (1:500 dilution, JI111605144, Jackson ImmunoResearch) were used to target the *F. novicida* and GBP primary antibodies or FLAG-tag (DYKDDDDK) antibody, respectively. Cells were counterstained in DAPI mounting medium (H-1200, Vecta Labs). Bacteria and GBPs were visualized and imaged using a Zeiss LSM 800 confocal. All immunofluorescence data was collected and analyzed using ZEN v3.2 (Blue edition).

### Peptide analysis and generation

The sequence of GBP1 (CCDS38658.1 [https://www.ncbi.nlm.nih.gov/CCDS/CcdsBrowse.cgi?REQUEST = CCDS&DATA = CCDS38658]) was analyzed for regions of antimicrobial potential using several bioinformatic tools including Collection of Anti-Microbial Peptides (CAMP) with support vector machine classifier^72^ and a window size of 20 amino acids. The net charge of these 20 amino acids, corresponding to that in CAMP analysis, was calculated. Putative antimicrobial peptides (Supplementary Table 5) were generated using solid-phase peptide synthesis to a minimum of 80% purity (Schaefer-N). The mouse GBP1 structure was predicted using SWISS-MODEL with human GBP1 (1DG3 [https://www.rcsb.org/structure/1dg3]) as the template. The predicted structure of GBP1 was visualized using PyMOL Molecular Graphics System v2.3.4.

### Antimicrobial assays

For bacterial viability assays, overnight cultures of bacteria were washed and resuspended with PBS to a concentration of 1 × 10^6^ CFU/mL respectively. Bacteria were then treated with solvent control, GBP peptides or GBP protein at the desired concentration and incubated at 37 ^o^C for 6 h. The O.D. of treated bacteria were read using the Tecan infinite 2000 pro and data was collected using i-Control software. Treated bacteria were serially diluted, plated onto TSB agar plates supplemented with 0.2% L-cysteine, LB, or BHI agar plates and incubated overnight at 37 ^o^C.

For bacterial membrane permeabilization assays, an overnight culture of bacteria was washed and resuspended with PBS to a concentration of 1 × 10^9^ CFU/mL. Bacteria were then treated with solvent control or peptides at the desired concentration and incubated at 37 ^o^C for 12 h. After washing with PBS, bacteria were stained with SYTOX Green (5 μM; S7020; Life Technologies) followed by washing with PBS and fixing in 4% PFA (20 min, room temperature). The fluorescence intensity for individual bacteria were measured by flow cytometry. Flow cytometry data was collected using BD FACSDiva and analyzed using FlowJo v10.7.

For analysis of DNA release, 1 × 10^9^ CFU/mL of bacteria were stained with Hoechst 33342 (10 μg/mL) and then treated with GBP1^28–67^ (10 μg/mL), solvent control (H_2_O) or 10% Triton X-100 for 4 h. The amount of released DNA in the supernatant was quantitated by measuring RFU at 440 nm on a Tecan infinite 2000 pro with excitation at 350 nm and data was collected using i-Control software.

### Peptide and protein binding assays

For FITC-GBP1^28–67^ peptide binding assay, an overnight culture of bacteria was washed and resuspended with PBS to a concentration of 1×10^9^ CFU/mL. Bacteria were then treated with 10 μg/mL of FITC-GBP1^28–67^ or FITC-control peptide for 1 h or 6 h at room temperature. Alternatively, bacteria were treated with 40 µM of mGBP1 protein or solvent control for 6 h. After washing with PBS, the relative fluorescence unit (RFU) of FITC at 520 nm was measured on a Tecan infinite 2000 pro with excitation at 480 nm and data was collected using i-Control software. For microscopy analysis of peptide binding, FITC-peptide-treated samples were stained with SYTOX Red (5 μM; S34859; Invitrogen) and Hoechst 33342 (10 μg/mL; H3570, Invitrogen) whereas mGBP1 treated bacteria were also stained with anti-His antibody (1:500 dilution, 2365 S, Cell Signaling Technology). Bacteria were visualized using Zeiss LSM 800 with Airyscan. Alternatively, FITC-peptide-treated samples were stained with SYTOX Red (5 µM) and the fluorescence intensity for individual bacteria were measured by flow cytometry. Flow cytometry data was collected using BD FACSDiva and analyzed using FlowJo v10.7.

For FITC-GBP1^28–67^ peptide removal assay, cultures of *F. novicida* were prepared as mentioned above. FITC-GBP1^28–67^-treated *F. novicida* were washed using 1 M of NaCl, 0.08% sarcosyl, 0.01% saponin (8047-15-2, Alfa Aesar) or PBS. The amount of remaining FITC signal on bacteria at 520 nm was measured on a Tecan infinite 2000 pro with excitation at 480 nm and data was collected using i-Control software.

### Scanning electron microscopy

Mid-logarithmic phase bacteria were washed and resuspended in PBS before peptide treatment at 100 µg/mL for 12 h or protein treatment at 1.84 µM for 6 h on coverslips. Treated bacteria were washed with PBS and post-fixed with 2.5% glutaraldehyde in 0.1 M phosphate buffer for 3 h and further washed with PBS. Cells were stained in 1% osmium tetroxide in distilled water for 1 h and dehydrated in a series of alcohol. Dehydrated samples were dried using liquid carbon dioxide critical point drying. Samples were then sputter-coated with platinum (3 nm thickness) at 15 mA for 2 min using the EMI TECH K550 Sputter coater and visualized under a Zeiss UltraPlus Field emission scanning electron microscope at 5 kV.

### Transmission electron microscopy

To visualize peptide-induced bacteriolysis, peptide-treated *F. novicida* were visualized under TEM. Briefly, peptide-treated bacteria were fixed with 2.5% glutaraldehyde in 0.1 M phosphate buffer for 3 h and washed with PBS. Fixed bacteria were stained with 1% osmium tetroxide in distilled water for 1 h and dehydrated in a series of alcohol. Samples were then absorbed onto carbon-coated TEM grids and stained with 2% UA before visualization on a JEOL 2100 F transmission electron microscope at 200 kV. Alternatively, samples were embedded in Procure 812 (C038, ProSciTech) and polymerized in a 60 °C oven overnight. Thin sections were cut at 80 nm, post-section stained with 2% UA and viewed using a HITACHI 7100 or a Zeiss Crossbeam 550 at 100 kV and 30 kV, respectively.

### Animal infection

*F. novicida* strain EXO186 was grown as described above. For survival and weight change analyses, mice were injected subcutaneously with 1.2 × 10^6^ colony-forming units (CFU) of *F. novicida* in 200 µL PBS. To assess bacterial burden, mice were injected subcutaneously with 6 × 10^5^ CFU of *F. novicida* in 200 µL PBS. After 3 days, liver and spleen were harvested and homogenized in PBS with metal beads for 2 min using the Qiagen TissueLyser II apparatus. *F. novicida* CFU were determined by plating lysates onto TSB agar supplemented with 0.2% L-cysteine and incubated overnight at 37 ^o^C. No randomization or blinding was performed.

### Statistical analysis

The GraphPad Prism 9.0 software was used for data analysis. Data are shown as mean ± s.e.m. Statistical significance was determined by *t* tests (two-tailed) for two groups or one-way analysis of variance (with Dunnett’s or Tukey’s multiple-comparisons test) for three or more groups. Survival curves were compared using the log-rank test. *P* < 0.05 was considered statistically significant.

### Reporting summary

Further information on research design is available in the Nature Research Reporting Summary linked to this article.

## Supplementary information

Supplementary Information

Reporting Summary

## Data Availability

All other data supporting the findings of this study are available in the article and its Supplementary files or from the corresponding author upon reasonable request. All unique biological materials generated in this study are available from the corresponding author. Source data are provided with this paper.

## Source data

Source Data
