# Supplementary material for: Pathogen-selective killing by guanylate-binding proteins as a molecular mechanism leading to inflammasome signaling
Source: Nat Commun. 2022 Jul 29;13:4395. doi: 10.1038/s41467-022-32127-0 (PMC9338265; doi:10.1038/s41467-022-32127-0)
Supplement: Supplementary file 1 — Supplementary Information [file 41467_2022_32127_MOESM1_ESM.docx]

**Supplementary Information**

**Inflammasome signaling requires pathogen-selective killing by guanylate-binding proteins**

Shouya Feng^1†^, Daniel Enosi Tuipulotu^1†^, Abhimanu Pandey^1^, Weidong Jing^1^, Cheng Shen^1^, Chinh Ngo^1^, Melkamu B. Tessema^2,3^, Fei-Ju Li^1^, Daniel Fox^1^, Anukriti Mathur^1^, Anyang Zhao^1^, Runli Wang^1^, Klaus Pfeffer^4^, Daniel Degrandi^4^, Masahiro Yamamoto^5,6^, Patrick C. Reading^2,3^, Gaetan Burgio^1^, Si Ming Man^1^*

^†^ These authors contributed equally: Shouya Feng, Daniel Enosi Tuipulotu

*Correspondence: siming.man@anu.edu.au

**SUPPLEMENTARY FIGURES**

**Supplementary Figure 1. GBPs contribute to activation of the AIM2 inflammasome by *F. novicida*.**

(**a**) Immunoblot analysis of caspase-1 (Casp-1) and gasdermin D (GSDMD) in WT, *Gbp*^chr3^-KO, *Ifnar1*^–/–^ or *Aim2*^–/–^ BMDMs left untreated (Med.) or assessed after infection with *F. novicida* (MOI 100) for 10 h. (**b**) The release of IL-1β, IL-18 and LDH from BMDMs after treatment as in **a**. (**c**) Confocal microscopy analysis of ASC specks (red) in WT, *Gbp1*^–/–^, *Gbp3*^–/–^ or *Aim2*^–/–^ BMDMs left untreated or assessed 10 h after infection with *F. novicida* (MOI 100) or 4 h after transfection with poly(dA:dT) (top). Quantitation of ASC inflammasome specks (bottom). At least 200 BMDMs were analyzed from each genotype. White arrows indicate ASC specks. (**d**) Light microscopy analysis of WT, *Gbp1*^–/–^, *Gbp3*^–/–^ or *Aim2*^–/–^ BMDMs left untreated (Med.) or following infection with *L. monocytogenes* (MOI 100) for 20 h, infection with MCMV (MOI 10) for 10 h or transfection of poly(dA:dT) (5 µg/ml) and pcDNA (5 µg/mL) for 4 h**.** White arrows indicate pyroptotic cells. Scale bars, 20 µm (**c** and **d**). ns, no statistical significance, ** *P*<0.01; *** *P*<0.001, **** *P*<0.0001, one-way ANOVA with Dunnett’s multiple-comparisons test (**b**-**c**). Data are pooled from three independent repeats (**b**-**c**, mean and s.e.m in **b**-**c**) or representative of three independent experiments (**d**). Source data are provided as a Source data file.

**Supplementary Figure 2. Generation of GBP1, GBP2, GBP3, GBP5, GBP7 and Pyrin knockout mice.**

(**a**) Schematic of the targeted exon of *Gbp1* in *Gbp1*^–/–^ mouse strain. (**b**) Immunoblot analysis of GBP1 (top) and β-actin (bottom) in WT or *Gbp1*^–/–^ colon tissues. (**c**) Schematic of the targeted exons of *Gbp2* in *Gbp2*^–/–^ mouse strain. (**d**) Immunoblot analysis of GBP2 (top) and β-actin (bottom) in WT or *Gbp2*^–/–^ colon tissues. (**e**) Schematic of the targeted exons of *Gbp3* in *Gbp3*^–/–^ mouse strain. (**f**) Immunoblot analysis of GBP3 (top) and β-actin (bottom) in WT or *Gbp3*^–/–^ colon tissues. (**g**) Schematic of the targeted exons of *Gbp5* in *Gbp5*^–/–^ mouse strain. (**h**) Immunoblot analysis of GBP5 (top) and β-actin (bottom) in WT or *Gbp5*^–/–^ colon tissues. (**i**) Schematic of the targeted exons of *Gbp7* in *Gbp7*^–/–^ mouse strain. (**j**) Immunoblot analysis of GBP7 (top) and β-actin (bottom) in WT or *Gbp7*^–/–^ colon tissues**.** (**k**) Schematic of the targeted exon of *Mefv* in *Mefv*^–/–^ mouse strain. (**l**) Immunoblot analysis of Pyrin (top) and β-actin (bottom) in WT or *Mefv*^–/–^ BMDMs left untreated (Med.) or assessed after LPS treatment. All loading controls (β-actin) were run on a separate blot. Blue asterisks (*) indicate gRNA targeted exons. Coding DNA (c); protein (p); deletion (del); frame shift (fs); termination (ter). Data are representative of three independent experiments (**b, d, f, h, g, j, l**). Source data are provided as a Source data file.

**Supplementary Figure 3. GBP1 and GBP3 do not affect the global production of pro-inflammatory cytokines or expression of other GBPs in response to *F. novicida* infection.**

(**a**) Immunoblot analysis of phospho-ERK (pERK), ERK, phospho-IκB (pIκB), IκB and β-actin in WT, *Gbp1*^–/–^ and *Gbp3*^–/–^ BMDMs 0-60 min after infection with *F. novicida* (MOI 100). (**b**) RT-PCR analysis of the genes encoding IL-1β, IL-6, IL-18, KC (*Cxcl1*), TNF and IFN-β in WT, *Gbp1*^–/–^ or *Gbp3*^–/–^ BMDMs left untreated or assessed 4 h after infection with *F. novicida* (MOI 100), relative to *Gapdh*. (**c**) Release of IL-6, KC, TNF and IFN-β in WT, *Gbp1*^–/–^ or *Gbp3*^–/–^ BMDMs after treatment as in **b**. (**d**) RT-PCR analysis of the genes encoding GBP2, GBP3, GBP5 and GBP7 in WT, *Gbp1*^–/–^BMDMs 4 h after infection with *F. novicida* (MOI 100), relative to *Gapdh*. (**e**) RT-PCR analysis of the genes encoding GBP1, GBP2, GBP5 and GBP7 in WT, *Gbp3*^–/–^ BMDMs 4 h after infection with *F. novicida* (MOI 100), relative to *Gapdh*. (**f**) Immunoblot analysis of GBP1, GBP2, GBP3, GBP5 and β-actin in WT, *Gbp1*^–/–^ and *Gbp3*^–/–^ BMDMs 0-20 h after infection with *F. novicida* (MOI 25). All loading controls (β-actin) were run on a separate blot. ns, no statistical significance, one-way ANOVA with Dunnett’s multiple-comparisons test (**b, c**) or two-tailed *t*-test (**d, e**). Data are representative of three independent experiments (**a, f**) or pooled from three independent repeats (**b**-**e**, mean and s.e.m in **b**-**e**). Source data are provided as a Source data file.

**Supplementary Figure 4. GBP1 and GBP3 are not required for the activation of the non-canonical NLRP3 inflammasome.**

(**a**) Immunoblot analysis of caspase-1 (Casp-1) and gasdermin D (GSDMD) in WT, *Gbp1*^–/–^, *Gbp2*^–/–^, *Nlrp3*^–/–^ or *Casp11*^–/–^ BMDMs left untreated (Med.) or assessed after infection with *C. rodentium* (MOI 20), *E. coli* (MOI 25) for 20 h or transfection of *E. coli* LPS (10 μg/ml) for 4 h. (**b**) The release of IL-1β, IL-18, TNF and LDH from BMDMs after treatment as in **a**. (**c**) Immunoblot analysis of Casp-1 and GSDMD in WT, *Gbp2*^–/–^, *Gbp3*^–/–^, *Nlrp3*^–/–^ or *Casp11*^–/–^ BMDMs treated as in **a**. (**d**) The release of IL-1β, IL-18, TNF and LDH from BMDMs after treatment as in **C**. Each symbol represents an independent experiment (**b**, **d**). ns, no statistical significance, * *P*<0.05, ** *P*<0.01; *** *P*<0.001, **** *P*<0.0001, one-way ANOVA with Dunnett’s multiple-comparisons test (**b**, **d**). Data are representative of three independent experiments (**a** and **d**) or pooled from three independent experiments (**b** and **d**; mean and s.e.m. in **b**, **d**). Source data are provided as a Source data file.

**Supplementary Figure 5. GBP1 and GBP3 are not required for the activation of the NLRC4, canonical NLRP3 and Pyrin inflammasomes.**

(**a, c**) Immunoblot analysis of caspase-1 (Casp-1) and gasdermin D (GSDMD) in WT, *Gbp1*^–/–^, *Gbp3*^–/–^ and *Nlrc4*^–/–^ BMDMs left untreated (Med.) or assessed after infection with *Salmonella enterica serovar* Typhimurium (*S.* Tm, MOI 2) for 4 h. (**b, d**) The release of IL-1β, IL-18 and LDH from BMDMs after treatment as in **a, c**. (**e, g**) Immunoblot analysis of Casp-1 and GSDMD of LPS-primed WT, *Gbp1*^–/–^, *Gbp3*^–/–^ and *Nlrp3*^–/–^ BMDMs left untreated (Med.) or assessed 4 h after stimulation with ATP (5 mM) or nigericin (Nig., 10 μM). (**f, h**) The release of IL-1β, IL-18 and LDH from BMDMs after treatment as in **e, g**. (**i, k**) Immunoblot analysis of Casp-1 and GSDMD of LPS-primed WT, *Gbp1*^–/–^, *Gbp3*^–/–^ and *Mefv*^–/–^ BMDMs left untreated (Med.) or assessed 20 h after stimulation with the supernatant of *C. difficile* (*C. dif.* Sup.). (**j, l**) The release of IL-1β, IL-18 and LDH from BMDMs after treatment as in **i, k**. Each symbol represents an independent experiment in **b**, **d**, **f**, **h**, **j**, **l**. ns, no statistical significance, one-way ANOVA with Dunnett’s multiple-comparisons test (**b**, **d**, **f**, **h**, **j**, **l**). Data are representative of three independent experiments (**a**, **c**, **e**, **g**, **I**, **k**) or pooled from three independent experiments (**b**, **d**, **f**, **h**, **j**, **l**; mean and s.e.m. in **b**, **d**, **f**, **h**, **j**, **l**). Source data are provided as a Source data file.

**Supplementary Figure 6. GBP2 and GBP5 recruitment to *F. novicida* does not require GBP1 and GBP3.**

(**a**) Confocal microscopy analysis of GBP2 (red) or GBP5 (red) and *F. novicida* (green) in WT, *Gbp1*^–/–^*, Gbp2*^–/–^, *Gbp3*^–/–^ and *Gbp5*^–/–^ BMDMs left untreated (Med.) or assessed 20 h after infection with *F. novicida* (MOI 20). White arrows indicate bacteria colocalized with GBP. (**b**) Confocal microscopy analysis of GBP7 (red) and *F. novicida* (green) in WT and *Gbp7*^–/–^ BMDMs left untreated (Med.) or assessed 20 h after infection with *F. novicida* (MOI 20). (**c**) Confocal microscopy analysis of FLAG-OVA, FLAG-GBP1, FLAG-GBP2, FLAG-GBP3, FLAG-GBP5 and FLAG-GBP7 (red) and *F. novicida* (green) in LA-4 cells left untreated (Med.), or 12 h after infection with *F. novicida* (MOI 100) primed with IFN-γ (100 U/ml). White arrows indicate bacteria colocalized with GBP. Scale bars, 10 µm (**a, b**) and 5 µm (**c**). Data are representative of three independent experiments (**a**-**b**).

**Supplementary Figure 7. Overexpression of individual FLAG-tagged GBPs does not facilitate recruitment of IRGB10 to GBP-coated *F. novicida*.**

(**a**) Confocal microscopy analysis of FLAG-OVA, FLAG-GBP1, FLAG-GBP2, FLAG-GBP3, FLAG-GBP5 and FLAG-GBP7 (red) and *F. novicida* (green) in LA-4 cells left untreated (Med.), or 12 h after infection with *F. novicida* (MOI 100) primed with or without IFN-γ (100 U/ml). (**b**) Confocal microscopy analysis of IRGB10 (red), FLAG-OVA, FLAG-GBP1, FLAG-GBP2, FLAG-GBP3, FLAG-GBP5 and FLAG-GBP7 (magenta) and *F. novicida* (green) in LA-4 cells left untreated (Med.), or 12 h after infection with *F. novicida* (MOI 100) primed with IFN-γ (100 U/ml). Scale bars, 5 µm (**a**) and 10 µm (**b**). Data are representative of three independent experiments (**a**-**b**).

**Supplementary Figure 8. Mouse GBP1 mediates bacteriolysis of *F. novicida.***

(**a**) Viability of *F. novicida* (*F. nov.*) and *E. coli* [as percentage of CFU in relation to solvent control (Sol.Ctrl.)] assessed 6 h after incubation with mGBP1 protein at 0.4, 4 or 40 μM. (**b**) Confocal microscopy analysis of Hoechst-stained total bacteria (blue), mGBP1 (green) and SYTOX (red) in *F. novicida* (*F. nov.*) and *E. coli* treated with 40 μM mGBP1 protein or solvent control for 6 h. White arrows indicate dead bacteria covered with mGBP1 protein. (**c**) Scanning electron microscopy analysis of the morphology of *F. novicida* 6 h after treatment with dialysis buffer (solvent control) or 1.84 μM of mGBP1 protein. Scale bars, 10 µm (**b**) and 200 nm (**c**). ns, no statistical significance; **** *P*<0.0001, one-way ANOVA with Dunnett’s multiple-comparisons test (**a**). Data are representative of three independent experiments (**a**-**c,** mean and s.e.m. in **a**). Source data are provided as a Source data file.

**Supplementary Figure 9. GBP1 peptide specifically kills *F. novicida* but not mammalian cells.**

(**a**) Flow cytometric plots showing the gating strategy for analysis of SYTOX positive singlets. (**b**) Viability of *E. coli* [as percentage of CFU in relation to solvent control (Sol.Ctrl.)] assessed 6 h after incubation with GBP1^28-67^ or WLBU2 at 0.1, 1 or 10 μg/ml. (**c**) Quantitation of FITC-GBP1^28-67^ bound to *F. novicida* after treatment with 1 M NaCl, 0.01% saponin or 0.08% sarcosyl (in relative fluorescence units, RFU). (**d**) Quantitation of the florescent signal of FITC-GBP1^28-67^ incubated with 1 M NaCl, 0.01% saponin or 0.08% sarcosyl. (**e**) Viability of *F. novicida* (as percentage of CFU in relation to untreated control) after incubation with 1 M NaCl, 0.01% saponin or 0.08% sarcosyl. (**f**) Viability of *F. novicida* (*F. nov.*, log_10_CFU) assessed 0, 0.5, 1, 2, 4 and 6 h after treatment with solvent control, 10 μg/ml of mGBP1 or WLBU2. (**g**) Viability of *F. novicida* [*F. nov.*, as percentage of CFU in relation to solvent control] assessed 6 h after incubation in PBS, saline or RPMI with GBP1^28-67^ or WLBU2 at 10 μg/ml. (**h**) Cell death of WT BMDMs, Vero, HEK293T and HT-29 cells after treatment with Sol.Ctrl., GBP1^28-67^, GBP1^209-238^, GBP1^424-452^, GBP1^558-577^ or WLBU2 at 10 μg/ml or 10% Triton X-100, as determined by the IncuCyte live-imaging system. (**i**) Cell death of WT BMDMs, Vero, HEK293T and HT-29 cells after treatment with Sol.Ctrl., GBP1^28-67^, GBP1^209-238^, GBP1^424-452^, GBP1^558-577^ or WLBU2 at 10 μg/ml for 24 h or treatment with 10% Triton X-100, as determined by percentage of LDH release. ns, no statistical significance; * *P*<0.05, *** *P*<0.001; **** *P*<0.0001, one-way ANOVA with Dunnett’s multiple-comparisons test (**b-g**). Data are representative of three independent experiments (**a**-**i**; mean and s.e.m. in **b-i**). Source data are provided as a Source data file.

**Supplementary Figure 10. GBP1 peptide mediates pathogen-selective killing of bacteria**

(**a**) Viability of *F. novicida* (*F. nov.*), *C. rodentium* (*C. rod.*), *E. coli*, *N. meningitidis* (*N. men.*), *P. aeruginosa* (*P. aer.*), *S. flexneri* (*S. flex.*), *S.* Typhimurium (*S.* Tm), *B. cereus* (*B. cer*.), *L. monocytogenes* (L. mono.) and *S. aureus* (*S. aur.*) [as percentage of CFU in relation to solvent control (Sol.Ctrl.)] assessed 6 h after incubation with GBP1^28-67^ or WLBU2 at 10 μg/ml. (**b**) Confocal microscopy analysis of Hoechst-stained total bacteria (blue), FITC-GBP1^28-67^ (green) and SYTOX (red) in *N. meningitidis* treated with 10 µg/mL FITC-GBP1^28-67^ or FITC-control peptide for 6 h. White arrows indicate dead bacteria covered with FITC-GBP1^28-67^. (**c**) Quantitation of FITC-GBP1^28-67^ bound to *N. meningitidis* and *E. coli* after 1 h incubation with 10 µg/mL of either FITC- GBP1^28-67^ or a FITC-control peptide (in relative fluorescence units, RFU). (**d**) Viability of *N. meningitidis* (*N. men.*) [as percentage of CFU in relation to solvent control (Sol.Ctrl.)] assessed 6 h after incubation with mGBP1 protein at 0.4, 4 or 40 μM. (**e**) Confocal microscopy analysis of Hoechst-stained total bacteria (blue), mGBP1 (green) and SYTOX (red) in *N. meningitidis* treated with solvent control or 40 μM mGBP1 for 6 h. White arrows indicate dead bacteria covered with mGBP1. (**f**) Viability of *N. meningitidis* (*N. men.*) and Δ*IpxA* mutant [as percentage of O.D. in relation to solvent control (Sol.Ctrl.)] assessed 6 h after incubation with mGBP1 protein at 0.4, 4 or 40 μM. Scale bars, 5 µm (**b**), 10 µm (**e**). ns, no statistical significance; ** *P*<0.01, *** *P*<0.001, **** *P*<0.0001, one-way ANOVA with Dunnett’s multiple-comparisons test (**a, d** and **f**), two-tailed *t*-test (**c**). Data are representative of three independent experiments (**a-f,** mean and s.e.m. in **a,** **c, d** and **f**). Source data are provided as a Source data file.

**Supplementary Figure 11. The GBP1^28-67^ peptide sequence is conserved amongst murine and human GBPs.**

(**a**) Alignment of the killing region from murine and human GBPs and the secondary structures. (**b**) Viability of *F. novicida* (*F. nov.*) and *E. coli* [as percentage of CFU in relation to solvent control (Sol.Ctrl.)] assessed 6 h after incubation with GBP3^22-61^ at 0.1, 1 or 10 μg/ml. (**c**) Scanning electron microscopy (top) and negative-stain transmission electron microscopy (bottom) analysis of the morphology of *F. novicida* 12 h after treatment with solvent control, 100 μg/ml of GBP3^22-61^. (**d**) Diagram illustrating the tertiary structure and surface accessibility of GBP1^28-67^ in within the predicted mouse GBP1 structure. (**e**) Viability of *F. novicida* (*F. nov.*), *E. coli* and *N. meningitidis* (*N. men.*) [as percentage of CFU in relation to solvent control (Sol.Ctrl.)] assessed 6 h after incubation with GBP1^28-67^, GBP1^28-38^, GBP1^38-67^ or GBP1^46-67^ at 10 μg/ml. (**f**) Quantitation of DNA released from *F. novicida* or *E. coli* following 4 hours incubation of 10 µg/mL of either solvent control (Sol. Ctrl.) or GBP1^28-67^. Scale bar, 200 nm (**c**). ns, no statistical significance; *** *P*<0.001; **** *P*<0.0001, one-way ANOVA with Dunnett’s multiple-comparisons test (**b** and **e**), two-tailed *t*-test (**f**). Data are representative of three independent experiments (**b**, **c** and **e-f**, mean and s.e.m. in **b, e** and **f**). Source data are provided as a Source data file.

**Supplementary Figure 12. GBPs are not required for the cytosolic entry of *F. novicida*.**

(**a**) Diagram illustrating the cytosolic escape assay. (**b**) Immunoblot analysis of caspase-1 (Casp-1) and gasdermin D (GSDMD) in WT, *Gbp1*^–/–^, *Gbp2*^–/–^, *Gbp3*^–/–^, *Gbp5*^–/–^, *Aim2*^–/–^, *Aim2*^–/–^*Nlrp3*^–/–^ or *Casp11*^–/–^ BMDMs left untreated (Med.), or assessed after infection with *F. novicida* (MOI 100) with or without LPS for 10 h or transfection with LPS (Trans. LPS) for 4 h. (**c**) The release of IL-1β, IL-18, TNF and LDH from BMDMs after treatment as in **b** in addition to stimulation with LPS only. ns, no statistical significance; **** *P*<0.0001, one-way ANOVA with Dunnett’s multiple-comparisons test (**c**). Data are representative of three independent experiments (**b**) or pooled from three independent experiments (**c**; mean and s.e.m. in **c**). Source data are provided as a Source data file.

**SUPPLEMENTARY TABLES**

**Supplementary Table 1:** Nucleotide sequence similarity (%) of *Gbp2* exons between WT, *Gbp1*^­–/–^ and *Gbp3*^–/–^ mice.

|  |  | **WT mice** | | | | | | | | | |
| --- | --- | --- | --- | --- | --- | --- | --- | --- | --- | --- | --- |
|  | ***Gbp2 e*xon** | **2** | **3** | **4** | **5** | **6** | **7** | **8** | **9** | **10** | **11** |
| ***Gbp1* ^–/–^ mice** | **2** | 100 |  |  |  |  |  |  |  |  |  |
|  | **3** |  | 100 |  |  |  |  |  |  |  |  |
|  | **4** |  |  | 100 |  |  |  |  |  |  |  |
|  | **5** |  |  |  | 100 |  |  |  |  |  |  |
|  | **6** |  |  |  |  | 100 |  |  |  |  |  |
|  | **7** |  |  |  |  |  | 100 |  |  |  |  |
|  | **8** |  |  |  |  |  |  | 100 |  |  |  |
|  | **9** |  |  |  |  |  |  |  | 100 |  |  |
|  | **10** |  |  |  |  |  |  |  |  | 100 |  |
|  | **11** |  |  |  |  |  |  |  |  |  | 100 |
| ***Gbp3* ^–/–^ mice** | **2** | 100 |  |  |  |  |  |  |  |  |  |
|  | **3** |  | 100 |  |  |  |  |  |  |  |  |
|  | **4** |  |  | 100 |  |  |  |  |  |  |  |
|  | **5** |  |  |  | 100 |  |  |  |  |  |  |
|  | **6** |  |  |  |  | 100 |  |  |  |  |  |
|  | **7** |  |  |  |  |  | 100 |  |  |  |  |
|  | **8** |  |  |  |  |  |  | 100 |  |  |  |
|  | **9** |  |  |  |  |  |  |  | 100 |  |  |
|  | **10** |  |  |  |  |  |  |  |  | 100 |  |
|  | **11** |  |  |  |  |  |  |  |  |  | 100 |

**Supplementary Table 2:** Nucleotide sequence similarity (%) of *Gbp5* exons between WT, *Gbp1*^­–/–^ and *Gbp3*^–/–^ mice.

|  |  | **WT mice** | | | | | | | | | |
| --- | --- | --- | --- | --- | --- | --- | --- | --- | --- | --- | --- |
|  | ***Gbp5 e*xon** | **2** | **3** | **4** | **5** | **6** | **7** | **8** | **9** | **10** | **11** |
| ***Gbp1* ^–/–^ mice** | **2** | 100 |  |  |  |  |  |  |  |  |  |
|  | **3** |  | 100 |  |  |  |  |  |  |  |  |
|  | **4** |  |  | 100 |  |  |  |  |  |  |  |
|  | **5** |  |  |  | 100 |  |  |  |  |  |  |
|  | **6** |  |  |  |  | 100 |  |  |  |  |  |
|  | **7** |  |  |  |  |  | 100 |  |  |  |  |
|  | **8** |  |  |  |  |  |  | 100 |  |  |  |
|  | **9** |  |  |  |  |  |  |  | 100 |  |  |
|  | **10** |  |  |  |  |  |  |  |  | 100 |  |
|  | **11** |  |  |  |  |  |  |  |  |  | 100 |
| ***Gbp3* ^–/–^ mice** | **2** | 100 |  |  |  |  |  |  |  |  |  |
|  | **3** |  | 100 |  |  |  |  |  |  |  |  |
|  | **4** |  |  | 100 |  |  |  |  |  |  |  |
|  | **5** |  |  |  | 100 |  |  |  |  |  |  |
|  | **6** |  |  |  |  | 100 |  |  |  |  |  |
|  | **7** |  |  |  |  |  | 100 |  |  |  |  |
|  | **8** |  |  |  |  |  |  | 100 |  |  |  |
|  | **9** |  |  |  |  |  |  |  | 100 |  |  |
|  | **10** |  |  |  |  |  |  |  |  | 100 |  |
|  | **11** |  |  |  |  |  |  |  |  |  | 100 |

**Supplementary Table 3:** Nucleotide sequence similarity (%) of *Gbp7* exons between WT and *Gbp2*^­–/–^ mice.

|  |  | **WT mice** | | | | | | | | | |
| --- | --- | --- | --- | --- | --- | --- | --- | --- | --- | --- | --- |
|  | ***Gbp7 e*xon** | **2** | **3** | **4** | **5** | **6** | **7** | **8** | **9** | **10** | **11** |
| ***Gbp2* ^–/–^ mice** | **2** | 100 |  |  |  |  |  |  |  |  |  |
|  | **3** |  | 100 |  |  |  |  |  |  |  |  |
|  | **4** |  |  | 100 |  |  |  |  |  |  |  |
|  | **5** |  |  |  | 100 |  |  |  |  |  |  |
|  | **6** |  |  |  |  | 100 |  |  |  |  |  |
|  | **7** |  |  |  |  |  | 100 |  |  |  |  |
|  | **8** |  |  |  |  |  |  | 100 |  |  |  |
|  | **9** |  |  |  |  |  |  |  | 100 |  |  |
|  | **10** |  |  |  |  |  |  |  |  | 100 |  |
|  | **11** |  |  |  |  |  |  |  |  |  | 100 |

**Supplementary Table 4:** Primers used in this study for *Gbp2* and *Gbp5* exon sequencing

| **Target** |  | **Sequence (5’-3’)** | **Reference** |
| --- | --- | --- | --- |
| *Gbp2*, Exon 2 | F | CCATACAGGGCTCCAAAACTC | This study |
|  | R | ACAGAAGGAACCAAGCACAG |  |
| *Gbp2*, Exon 3 | F | GTCCCATGCTCATTCTGCTT | This study |
|  | R | TAAATGCAGGAGTGGGGTGT |  |
| *Gbp2*, Exon 4 | F | GGGGACAGATAAAGGCCTTCT | This study |
|  | R | AGGACTATGCTAGAGAACACCA |  |
| *Gbp2*, Exon 5 | F | CACATTGATTTGGGGAGGCA | This study |
|  | R | ACCGGGCAGAGATGACATTT |  |
| *Gbp2*, Exon 6 | F | GCTTACCTAGGCTTTGCGAA | This study |
|  | R | AGGGAAGTCAAGGTGAGAAGT |  |
| *Gbp2*, Exon 7 | F | TCACTCTACTAACTGACAGCCA | This study |
|  | R | CATCCCTCAAATGGTCCATGT |  |
| *Gbp2*, Exon 8 | F | CACCTTTCAACCATCCTTGTGA | This study |
|  | R | ATTCCTCACCAAGCCCTCAA |  |
| *Gbp2*, Exon 9 | F | ACACATTGCCTTGTCTCCAAC | This study |
|  | R | CAAGAGCTTCCACCATCTGC |  |
| *Gbp2*, Exon 10 | F | TCATAATCCTTGCTTCCCCTCA | This study |
|  | R | TTGGGGCACAGGAATGTACT |  |
| *Gbp2*, Exon 11 | F | TGAGTTCAGCCTCCATTTCTG | This study |
|  | R | GTTAAAAGTTGCCTGTTCTGTGA |  |
| *Gbp5*, Exon 2 | F | ATTTCTTCCCCTGCAGAGAC | This study |
|  | R | TGGAGAAGACTCAGCAAGCA |  |
| *Gbp5*, Exon 3 | F | CATTCCTCTGCGTCCATTCC | This study |
|  | R | CAACTGGTGGTGAGAAGGGA |  |
| *Gbp5*, Exon 4 | F | GCCTCCCTGCAAATACTCTTG | This study |
|  | R | GCCATCTCCTTAGCTCCTGT |  |
| *Gbp5*, Exon 5 | F | ACCCTCATGCATGTCACTGA | This study |
|  | R | TGTAGAAGTGCTTGGAGGGG |  |
| *Gbp5*, Exon 6 | F | TGCAGTATCAGGGGAAAGGA | This study |
|  | R | TCTCTGTGTGTCCATTACTCTCT |  |
| *Gbp5*, Exon 7 | F | AATCACATAGCTCCTCGGGG | This study |
|  | R | TTGCCTTCTGTGACCCTGAT |  |
| *Gbp5*, Exon 8 | F | ACAACTTTCCCAGCTTCGAT | This study |
|  | R | TGTTCAGGCAGCAGAAGAGA |  |
| *Gbp5*, Exon 9 + 10 | F | TGAGCTTTTCAGGGGTTGTC | This study |
|  | R | CGGACTGTACTGAGGAGCC |  |
| *Gbp5*, Exon 11 | F | TGAGCTTTTCAGGGGTTGTC | This study |
|  | R | CGGACTGTACTGAGGAGCC |  |

**Supplementary Table 5:** Peptide sequences

| **Peptide** | **Sequence** |
| --- | --- |
| GBP1^28-67^ | LDILSAIQNPVVVVAIVGFYHTGKSYLMNKLAGKQKGFSL |
| GBP1^28-38^ | LDILSAIQNPV |
| GBP1^38-67^ | VVVVAIVGFYHTGKSYLMNKLAGKQKGFSL |
| GBP1^46-67^ | FYHTGKSYLMNKLAGKQKGFSL |
| GBP1^209-238^ | GTDENTKKFNMPRLCIRKFFPKRKCFIFDR |
| GBP1^424-452^ | TFYKPGGYYLFLQRKQELEKKYIQTPGKG |
| GBP1^558-577^ | LLKQGFQNESLQLRQEIEKI |
| FITC-GBP1^28-67^ | FITC-(Ahx)LDILSAIQNPVVVVAIVGFYHTGKSYLMNKLAGKQKGFSL |
| FITC-control | FITC-(Ahx)KLIKLLDRLASRSVS |
| GBP3^22-61^ | IRILEQIAQPLVVVAIVGLYRTGKSYLMNRLAGRNHGFSL |
| WLBU2 | RRWVRRVRRWVRRVVRVVRRWVRR |

**Supplementary Table 6:** Primers used in this study for qPCR

| **Target** |  | **Sequence (5’-3’)** | **Reference** |
| --- | --- | --- | --- |
| *Gbp1* | F | AAGAACATGCCTCCACCTCG | ^1^ |
|  | R | ATCCAAAGCTGTCCCCGAAG |  |
| *Gbp2* | F | CTGCACTATGTGACGGAGCTA | ^2^ |
|  | R | CGGAATCGTCTACCCCACTC |  |
| *Gbp3* | F | CCAGAAAACCAACTGGAACGGAA | ^3^ |
|  | R | TCTCCAGACAAGGCACAGTC |  |
| *Gbp5* | F | CTGAACTCAGATTTTGTGCAGGA | ^2^ |
|  | R | CATCGACATAAGTCAGCACCAG |  |
| *Gbp7* | F | TTGAGGAAATGCCAGAGGACCAGT | ^3^ |
|  | R | GTCTCCACTATTGATAGCATCCACG |  |
| *Il1b* | F | GACCTTCCAGGATGAGGACA | ^4^ |
|  | R | AGCTCATATGGGTCCGACAG |  |
| *Il18* | F | GCCTCAAACCTTCCAAATCA | ^4^ |
|  | R | TGGATCCATTTCCTCAAAGG |  |
| *Il6* | F | CAAGAAAGACAAAGCCAGAGTC | ^4^ |
|  | R | GAAATTGGGGTAGGAAGGAC |  |
| *Cxcl1* | F | CAATGAGCTGCGCTGTCAGTG | ^4^ |
|  | R | CTTGGGGACACCTTTTAGCATC |  |
| *Tnf* | F | CATCTTCTCAAAATTCGAGTGACAA | ^4^ |
|  | R | TGGGAGTAGACAAGGTACAACCC |  |
| *Ifnb* | F | GCCTTTGCCATCCAAGAGATGC | ^4^ |
|  | R | ACACTGTCTGCTGGTGGAGTTC |  |
| *Gapdh* | F | CGTCCCGTAGACAAAATGGT | ^4^ |
|  | R | TTGATGGCAACAATCTCCAC |  |

**SUPPLEMENTARY REFERENCES**

1 Sahay, B. *et al.* Induction of Interleukin 10 by Borrelia burgdorferi Is Regulated by the Action of CD14-Dependent p38 Mitogen-Activated Protein Kinase and cAMP-Mediated Chromatin Remodeling. *Infect Immun* **86**, doi:10.1128/IAI.00781-17 (2018).

2 Man, S. M. *et al.* The transcription factor IRF1 and guanylate-binding proteins target activation of the AIM2 inflammasome by Francisella infection. *Nat Immunol* **16**, 467-475, doi:10.1038/ni.3118 (2015).

3 Yamamoto, M. *et al.* A cluster of interferon-γ-inducible p65 GTPases plays a critical role in host defense against *Toxoplasma gondii*. *Immunity* **37**, 302-313, doi:10.1016/j.immuni.2012.06.009 (2012).

4 Man, S. M. *et al.* IRGB10 Liberates Bacterial Ligands for Sensing by the AIM2 and Caspase-11-NLRP3 Inflammasomes. *Cell* **167**, 382-396.e317, doi:10.1016/j.cell.2016.09.012 (2016).
