# Supplementary material for: Pathogen-selective killing by guanylate-binding proteins as a molecular mechanism leading to inflammasome signaling
Source: Nat Commun. 2022 Jul 29;13:4395. doi: 10.1038/s41467-022-32127-0 (PMC9338265; doi:10.1038/s41467-022-32127-0)
Supplement: Supplementary file 2 — Reporting Summary [file 41467_2022_32127_MOESM2_ESM.pdf]

nature portfolio

Corresponding author(s): Si Ming Man

Last updated by author(s): May 5, 2022

Reporting Summary

Nature Portfolio wishes to improve the reproducibility of the work that we publish. This form provides structure for consistency and transparency in reporting. For further information on Nature Portfolio policies, see our Editorial Policies and the Editorial Policy Checklist.

Statistics

For all statistical analyses, confirm that the following items are present in the figure legend, table legend, main text, or Methods section.

n/a	Confirmed	
☐	☒	The exact sample size (n) for each experimental group/condition, given as a discrete number and unit of measurement
☐	☒	A statement on whether measurements were taken from distinct samples or whether the same sample was measured repeatedly
☐	☒	The statistical test(s) used AND whether they are one- or two-sided<br>*Only common tests should be described solely by name; describe more complex techniques in the Methods section.*
☒	☐	A description of all covariates tested
☐	☐	A description of any assumptions or corrections, such as tests of normality and adjustment for multiple comparisons
☐	☒	A full description of the statistical parameters including central tendency (e.g. means) or other basic estimates (e.g. regression coefficient) AND variation (e.g. standard deviation) or associated estimates of uncertainty (e.g. confidence intervals)
☐	☐	For null hypothesis testing, the test statistic (e.g. F, t, r) with confidence intervals, effect sizes, degrees of freedom and P value noted<br>*Give P values as exact values whenever suitable.*
☒	☐	For Bayesian analysis, information on the choice of priors and Markov chain Monte Carlo settings
☒	☐	For hierarchical and complex designs, identification of the appropriate level for tests and full reporting of outcomes
☒	☐	Estimates of effect sizes (e.g. Cohen's d, Pearson's r), indicating how they were calculated

Our web collection on statistics for biologists contains articles on many of the points above.

Software and code

Policy information about availability of computer code

Data collection	IncuCyte v2018B<br>ImageLab Software v6.01<br>ZEN v3.2 (Blue Edition)<br>xPONENT v4.2<br>BD FACSDiva<br>i-Control<br>QuantStudio 12K Flex Real-time PCR system<br>Electron microscopy samples were imaged on a Zeiss UltraPlus Field Emission, Hitachi 7100 or Zeiss Crossbeam electron microscopes.
Data analysis	GraphPad PRISM 9.0, ImageLab Software v6.01, FlowJo v10.7, ZEN v3.2 (Blue Edition), PyMOL Molecular Graphics System v2.3.4

For manuscripts utilizing custom algorithms or software that are central to the research but not yet described in published literature, software must be made available to editors and reviewers. We strongly encourage code deposition in a community repository (e.g. GitHub). See the Nature Portfolio guidelines for submitting code & software for further information.

Data

Policy information about availability of data

All manuscripts must include a data availability statement. This statement should provide the following information, where applicable:

- Accession codes, unique identifiers, or web links for publicly available datasets
- A description of any restrictions on data availability
- For clinical datasets or third party data, please ensure that the statement adheres to our policy

The authors declare that all data supporting the findings of this study are available within the paper, the Supplementary Information and Source data file. All unique biological materials generated in this study are available from the corresponding author.

Field-specific reporting

Please select the one below that is the best fit for your research. If you are not sure, read the appropriate sections before making your selection.

[x] Life sciences   [ ] Behavioural & social sciences   [ ] Ecological, evolutionary & environmental sciences

For a reference copy of the document with all sections, see nature.com/documents/nr-reporting-summary-flat.pdf

Life sciences study design

All studies must disclose on these points even when the disclosure is negative.

Sample size	No statistical methods were used to predetermine sample sizes. Sample sizes were chosen according to accepted standards in the field and based on our experience with similar published studies (PMID:30531979, PMID:32029733, PPMID:25774715, PMID:27693356). Specific details of sample size and statistical analysis are presented in the figure legends.
Data exclusions	No data were excluded.
Replication	The experimental findings were reliably reproduced. See details in figure legends.
Randomization	For in vitro experiments, all cells were maintained in the same environment and were randomly assigned to the experimental groups.
Blinding	All results presented are purely based on objective analysis of the capture data, without subjective interpretation. Hence, blinding was not required for this study.

Reporting for specific materials, systems and methods

We require information from authors about some types of materials, experimental systems and methods used in many studies. Here, indicate whether each material, system or method listed is relevant to your study. If you are not sure if a list item applies to your research, read the appropriate section before selecting a response.

Materials & experimental systems

n/a	Involved in the study	
[ ]	[x]	Antibodies
[ ]	[x]	Eukaryotic cell lines
[x]	[ ]	Palaeontology and archaeology
[ ]	[x]	Animals and other organisms
[x]	[ ]	Human research participants
[x]	[ ]	Clinical data
[x]	[ ]	Dual use research of concern

Methods

n/a	Involved in the study	
[x]	[ ]	ChIP-seq
[ ]	[x]	Flow cytometry
[x]	[ ]	MRI-based neuroimaging

Antibodies

Antibodies used	Caspase-1 (1:3000 dilution, AG-20B-0042, Adipogen)<br>GSDMD (1:3000 dilution, ab209845, Abcam)<br>GBP1 (1:20 dilution, a gift from Dr. Eva Frickel)<br>GBP2 (1:1000 dilution, CAC07820, Biomatik)<br>GBP2 (1:500 dilution, a gift from Dr. Klaus Pfeffer and Dr. Daniel Degrandi)<br>GBP3 (1:1000 dilution, SA0035 RB1060, Biomatik)<br>GBP3 (1:20 dilution, SA0035 RB1059, Biomatik)<br>GBP5 (1:1000 dilution, a gift from Dr. Klaus Pfeffer and Dr. Daniel Degrandi)

GBP7 (1:1000 dilution, SA0039 RB1065, Biomatik)
GBP7 (1:200 dilution, a gift from Klaus Peffer and Dr. Daniel Degrandi)
Pyrin (1:100 dilution, 195975, Abcam)
pIkB (1:1000 dilution, 2859, Cell Signaling Technologies)
IkB (1:1000 dilution, 9242, Cell Signaling Technologies)
pERK (1:1000 dilution, 9101, Cell Signaling Technologies)
ERK (1:1000 dilution, 9102, Cell Signaling Technologies)
B-actin (1:10000 dilution, 8457, Cell Signaling Technologies)
Asc (1:5000 dilution, clone AL177, AG-25B-0006-C100, Adipogen)
Rhodamine red Anti-rabbit (1:500 dilution, 111295144, Jackson ImmunoResearch)
HRP-conjugated Anti-rabbit (1:5000 dilution, 111035144, Jackson ImmunoResearch)
HRP-conjugated Anti-mouse (1:5000 dilution, 111035146, Jackson ImmunoResearch)
Anti-Francisella (1:500 dilution, a gift from Dr. Denise Monack)
Alexa Fluor 488 Anti-chicken (1:500 dilution, 103545155, Jackson ImmunoResearch)
Alexa Fluor 647 Anti-rabbit IgG (1:500 dilution, 111605144, Jackson ImmunoResearch)
Anti-DYKDDDDK (1:200 dilution, 2368S, Cell Signaling Technologies)
Anti-His (1:500 dilution, 2365S, Cell Signaling Technology)

Validation

Validation of the following antibodies are available on the manufacturer's website:

Caspase-1 (https://adipogen.com/ag-20b-0042-anti-caspase-1-p20-mouse-mab-casper-1.html/)
GSDMD (https://www.abcam.com/gsdmd-antibody-epr19828-ab209845.html)
Pyrin (https://www.abcam.com/pyrin-antibody-epr18676-ab195975.html)
pIkB (https://www.cellsignal.com/products/primary-antibodies/phospho-ikba-ser32-14d4-rabbit-mab/2859)
IkB (https://www.cellsignal.com/products/primary-antibodies/ikba-antibody/9242)
pERK (https://www.cellsignal.com/products/primary-antibodies/phospho-p44-42-mapk-erk1-2-thr202-tyr204-antibody/9101)
ERK (https://www.cellsignal.com/products/primary-antibodies/p44-42-mapk-erk1-2-antibody/9102)
B-actin (https://www.cellsignal.com/products/primary-antibodies/b-actin-d6a8-rabbit-mab/8457)
Asc (https://adipogen.com/ag-25b-0006-anti-asc-pab-al177.html)
Rhodamine red Anti-rabbit (https://www.jacksonimmuno.com/catalog/products/111-295-144)
HRP-conjugated Anti-rabbit (https://www.jacksonimmuno.com/catalog/products/111-035-144)
HRP-conjugated Anti-mouse (https://www.jacksonimmuno.com/catalog/products/115-035-146)
Alexa Fluor 488 Anti-chicken (https://www.jacksonimmuno.com/catalog/products/103-545-155)
Alexa Fluor 647 Anti-rabbit IgG (https://www.jacksonimmuno.com/catalog/products/111-605-144)
Anti-DYKDDDDK (https://www.cellsignal.com/products/primary-antibodies/dykddddk-tag-antibody-binds-to-same-epitope-as-sigma-s-anti-flag-m2-antibody/2368)
Anti-His (https://www.cellsignal.com/products/primary-antibodies/his-tag-antibody/2365)
GBP2 (https://www.biomatik.com/antibodies/gbp2-polyclonal-antibody-cat-cac07820/)

Validation of gifted antibodies are available in specific publications:

GBP1 (a gift from Dr. Eva Frickel, PMID:21931713)
GBP2 (a gift from Dr. Klaus Pfeffer and Dr. Daniel Degrandi, PMID:18025219)
GBP5 (1:1000 dilution, a gift from Dr. Klaus Pfeffer and Dr. Daniel Degrandi, PMID:18025219)
GBP7 (1:200 dilution, a gift from Klaus Peffer and Dr. Daniel Degrandi, PMID:18025219)
Anti-Francisella (1:500 dilution, a gift from Dr. Denise Monack, PMID:16230474)

Validation of the following antibodies was performed in this manuscript:

GBP3 (1:1000 dilution, Generated by Biomatik under project SA0035 RB1060, used for western blotting)
GBP3 (1:20 dilution, Generated by Biomatik under project SA0035 RB1059, used for immunofluoresence)
GBP7 (1:1000 dilution, Generated by Biomatik under project SA0039 RB1065, used for western blotting)

Eukaryotic cell lines

Policy information about cell lines

Cell line source(s)

L-929 cell line was a kind gift from Prof. David Tscharke (Australian National University)
Vero cell line was a kind gift from Prof. David Tscharke (Australian National University)
HEK293T cell line was a kind gift from Prof. Carola Vinuesa (Australian National University)
HT-29 cell line was a kind gift from Dr. Nadeem Kaakoush (Uninversity of New South Wales)
LA-4 cell line was a kind gift from Prof. Patrick Reading (The University of Melbourne)
M2-10B4 cell line was sourced from the American Type Culture Collection (Cat#:CRL-1972)

Authentication

Cell lines were verified by the commercial supplier or by the donating laboratory. The identity of all cell lines were frequently checked by their morphological features.

Mycoplasma contamination

All cell lines were tested to be mycoplasma-negative by laboratories of origin or manufacturer.

Commonly misidentified lines (See ICLAC register)	No commonly misidentified cell lines are used in this study.

Animals and other organisms

Policy information about studies involving animals; ARRIVE guidelines recommended for reporting animal research

Laboratory animals	C57BL/66NcrlAnu mice and Mefv–/– mice were sourced from The Australian National University. Nlrp3–/– (PMID: 22753929) and Casp11–/– (PMID: 9491891) mice were sourced from The Jackson Laboratory. Nlrc4–/– mice (PMID: 15190255) were sourced from the University of Queensland. Aim2–/– mice (PMID:20457908) were sourced from Genentech. GBPchr3-KO mice (PMID: 22795875) were sourced from Osaka University. Aim2–/– Nlrp3–/– mice were generated by crossing Aim2–/– mice and Nlrp3–/– mice. All mice are on the C57BL/66NcrlAnu background, or backcrossed to C57BL/66NcrlAnu background for at least 10 generations.<br><br>Mouse cages receive 16 fresh HEPA-filtered air changes per hour within rooms kept at 18-22 degree Celsius and 30% humidity. A 12 hour light-dark cycle is automatically controlled in each room. Male and female mice between 6-8 weeks old were used for experiments.
Wild animals	No wild animals were used in the study.
Field-collected samples	No field-collected samples were used in the study.
Ethics oversight	Mice were bred and maintained at The Australian National University and all animal studies were conducted in accordance with the Protocol Number A2020/19 approved by The Australian National University Animal Experimentation Ethics Committee.

Note that full information on the approval of the study protocol must also be provided in the manuscript.

Flow Cytometry

Plots

Confirm that:

- [x] The axis labels state the marker and fluorochrome used (e.g. CD4-FITC).
- [x] The axis scales are clearly visible. Include numbers along axes only for bottom left plot of group (a 'group' is an analysis of identical markers).
- [x] All plots are contour plots with outliers or pseudocolor plots.
- [x] A numerical value for number of cells or percentage (with statistics) is provided.

Methodology

Sample preparation	An overnight culture of bacteria was washed and resuspended with PBS to a concentration of 1x10^9 CFU/mL. Bacteria were then treated with solvent control or peptides at the desired concentration and incubated at 37oC for 12 hours. After washing with PBS, bacteria were stained with SYTOX Green (5 µM; S7020; Life Technologies) followed by washing with PBS and fixing in 4% PFA (20 min, room temperature). The fluorescence intensity for individual bacteria were measured by flow cytometry and analyzed using FlowJo.<br><br>or<br><br>For FITC-GBP1 peptide binding assay, an overnight culture of bacteria was washed and resuspended with PBS to a concentration of 1x10^9 CFU/mL.Bacteria were then treated with 10 µg/ml of FITC-GBP128-67 or FITC-control peptide for 6 h at room temperature. After washing with PBS, peptide-treated samples were stained with SYTOX Red (5 µM; S34859; InvitrogenTM) followed by washing with PBS and fixing in 4% PFA (20 min, room temperature). The fluorescence intensity for individual bacteria were measured by flow cytometry and analyzed using FlowJo.
Instrument	BD LSRII
Software	BD FACSDiva was used to collect data. FlowJo v10.7 was used to analyse data.
Cell population abundance	Pure bacterial culture was used.
Gating strategy	FSC/SSC to SYTOX-Green for bacterial permeabilisation assays.<br><br>FSC/SSC to FITC vs. SYTOX Red for peptide binding assays.

- [x] Tick this box to confirm that a figure exemplifying the gating strategy is provided in the Supplementary Information.
